# A Highly Stable Yttrium Organic Framework as a Host for Optical Thermometry and D_2_O Detection

**DOI:** 10.1002/chem.202200410

**Published:** 2022-03-04

**Authors:** Thomas W. Chamberlain, Rafael V. Perrella, Tamires M. Oliveira, Paulo C. de Sousa Filho, Richard I. Walton

**Affiliations:** ^1^ Department of Chemistry University of Warwick Coventry CV4 7AL UK; ^2^ Institute of Chemistry University of Campinas PO Box 6154 13083-970 Campinas SP Brazil

**Keywords:** energy transfer, heavy water detections, lanthanides, luminescence thermometry, metal–organic frameworks

## Abstract

The yttrium organic framework (Y_0.89_Tb_0.10_Eu_0.01_)_6_(BDC)_7_(OH)_4_(H_2_O)_4_ (BDC=benzene‐1,4‐dicarboxylate) is hydrothermally stable up to at least 513 K and thermally stable in air in excess of 673 K. The relative intensities of luminescence of Tb^3+^ and Eu^3+^ are governed by Tb^3+^‐to‐Eu^3+^ phonon‐assisted energy transfer and Tb^3+^‐to‐ligand back transfer and are responsible for the differing temperature‐dependent luminescence of the two ions. This provides a ratiometric luminescent thermometer in the 288–573 K temperature range, not previously seen for MOF materials, with a high sensitivity, 1.69±0.04 % K^−1^ at 523 K. In aqueous conditions, loosely bound H_2_O can be replaced by D_2_O in the same material, which modifies decay lifetimes to yield a quantitative luminescent D_2_O sensor with a useful sensitivity for practical application.

## Graphical abstract

**A hydrothermally stable and thermally robust yttrium MOF** can be substituted with photoluminescent lanthanide ions to act as a ratiometric luminescent thermometer. Accurate temperature determination is possible up to at least 573 K, the highest of any MOF reported to date. Monitoring of the luminescent decay lifetime allows detection of D_2_O in water with a sensitivity suited for practical applications.

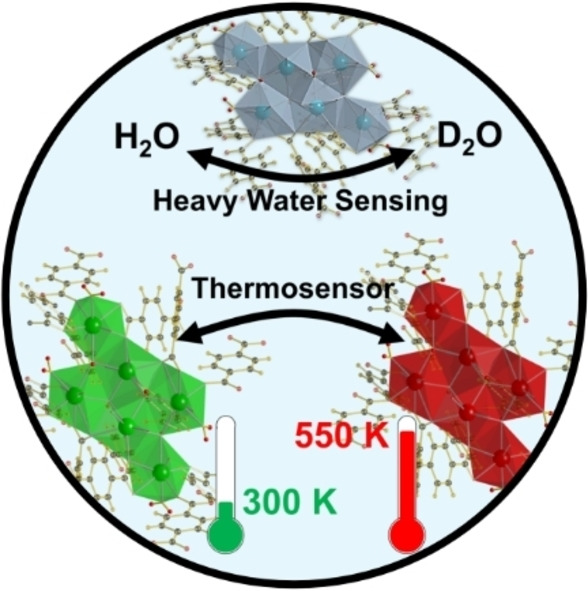

## Introduction

Temperature is a fundamental physical parameter and therefore it is vital in many situations that it can be accurately determined. A wide variety of thermometers have been developed for this purpose and are based on a range of temperature dependent physical properties, such as volume, electrical potential and electrical conductance.[^1^, ^2^, ^3^, ^4^] These more traditional temperature sensors rely on heat transfer from the substance to the sensor and must reach equilibrium before the temperature can be accurately measured. This makes these thermometers unsuitable for measurements on fast moving objects or on a small scale, for example, in living cells[^5^, ^6^, ^7^, ^8^] or in catalytical reactions in the mesoscopic or nanoscopic regime.^[9]^ This intrinsic drawback associated with conventional thermometers has driven research into novel thermometric methods that are non‐invasive and can work accurately on a small scale without the need for direct contact between the temperature sensor and the substrate.

One potential solution to this is the use of photoluminescent thermometers, which utilise a material's response to irradiated light in order to determine its precise temperature.^[10]^ In particular, monitoring the excitation ratio of Eu and Tb ions within a mixed metal lanthanoid (Ln) material has proven to be a reliable technique for measuring the temperature of a substrate. It is well known that Ln ions provide a range of advantageous luminescence properties to materials and complexes, such as long excited state lifetimes and narrow emission bandwidths resulting from electronic transitions within their 4f orbitals.[^11^, ^12^, ^13^] However, most 4f–4f transitions are electric dipole‐forbidden, resulting in poor light absorption and a very low molar absorption coefficient for many lanthanoid materials.[^14^, ^15^] Metal–organic frameworks (MOFs) are able to overcome this obstacle by exploiting strongly absorbing organic linkers that are able to greatly enhance 4f–4f transitions, known as the antenna effect, leading to much enhanced molar absorption coefficients and emission quantum yields.^[16]^

Mixed lanthanide‐based MOF thermometers have already been studied in recent years[^17^, ^18^, ^19^] and, although many of these materials show promising thermometric properties, a range of drawbacks still hold them back. In particular, narrow detection ranges, low sensitivities and low stability under irradiation severely limit the potential applications of all currently reported materials.^[20]^ In this regard, a highly stable ratiometric MOF thermometer, with good sensitivity over a wide temperature range, and in water or other solvents is needed. Herein, we report the synthesis of a highly stable yttrium benzene‐1,4‐dicarboxylate (BDC) MOF Y_6_(BDC)_7_(OH)_4_(H_2_O)_4_ (Y_6_‐MOF) as a host for Eu and Tb and evaluate its ratiometric photoluminescent activity over a wide temperature range in air and water.

**Figure 1:**
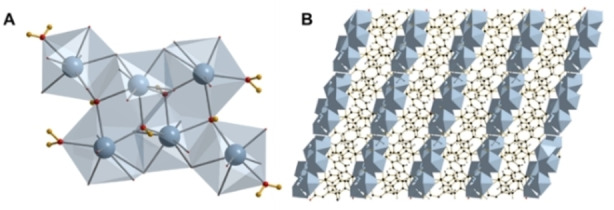
Structure of Y_6_‐MOF. (A): Single hexanuclear cluster highlighting μ_3_‐OH and terminally bound water molecules. Yttrium is shown in grey, oxygen in red and hydrogen in yellow. (B): Overall structure of Y_6_‐MOF, with Y_6_(OH)_4_(H_2_O)_4_ clusters depicted as grey polyhedra.

Y_6_‐MOF was first reported by Weng et al.,^[21]^ where the synthesis of a set of three novel rare‐earth‐based frameworks with isomorphous structures was described for Y, Yb and Er. It consists of hexanuclear clusters, Figure 1A, containing three distinct eight‐coordinate Y^III^ ions bridged by μ_3_‐OH groups with four of the Y centres terminally coordinated by water molecules. The hexameric clusters are held together by two distinct BDC linkers, one bidentate and one tridentate to form a structure containing one dimensional channels of occluded water, Figure 1B.

The Yb^III^ analogue of Y_6_‐MOF was later investigated by Burnett et al.^[22]^ as a bifunctional dual acid catalyst for the conversion of glucose into 5‐hydroxymethylfurfural. Here, Yb_6_‐MOF was noted for its extremely high stability, both thermally (*ca* 500 °C) and with no loss in crystallinity observed after heating hydrothermally in water at 240 °C for multiple days. Due to the exceptional stability of Yb_6_‐MOF, the spectroscopically inert Y^III^ analogue was targeted as the most suitable host material to avoid energy loss processes between metal centres.

## Results and Discussion

The synthesis method of Weng et al. required prehydrolysis of a rare‐earth salt for an unspecified time and precise adjustment of pH^[21]^ which made the synthesis difficult to reproduce. In contrast, the method of Burnett et al. for the ytterbium analogue required a two‐step synthesis via the MOF Yb_2_(BDC)_3_(DMF)_2_(H_2_O)_2_,^[22]^ itself first made in a mixed DMF/water solution.^[23]^ Hence we have developed a new synthetic route to (Y_0.89_Tb_0.10_Eu_0.01_)_6_‐MOF in which an aqueous solution of stoichiometric yttrium, europium and terbium precursors was heated hydrothermally along with the disodium salt of the linker under basic conditions. This yielded the desired material, which was then hydrothermally washed in water to yield phase pure (Y_0.89_Tb_0.10_Eu_0.01_)_6_‐MOF. The MOF became more crystalline over time whilst heating, forming large micron sized crystals after 72 h of heating (Figure S1). The same method was used to prepare samples of (Y_0.9_Tb_0.10_)_6_‐MOF and (Y_0.99_Eu_0.01_)_6_‐MOF as reference materials. Further synthesis details are provided in Supporting Information.

**Figure 2:**
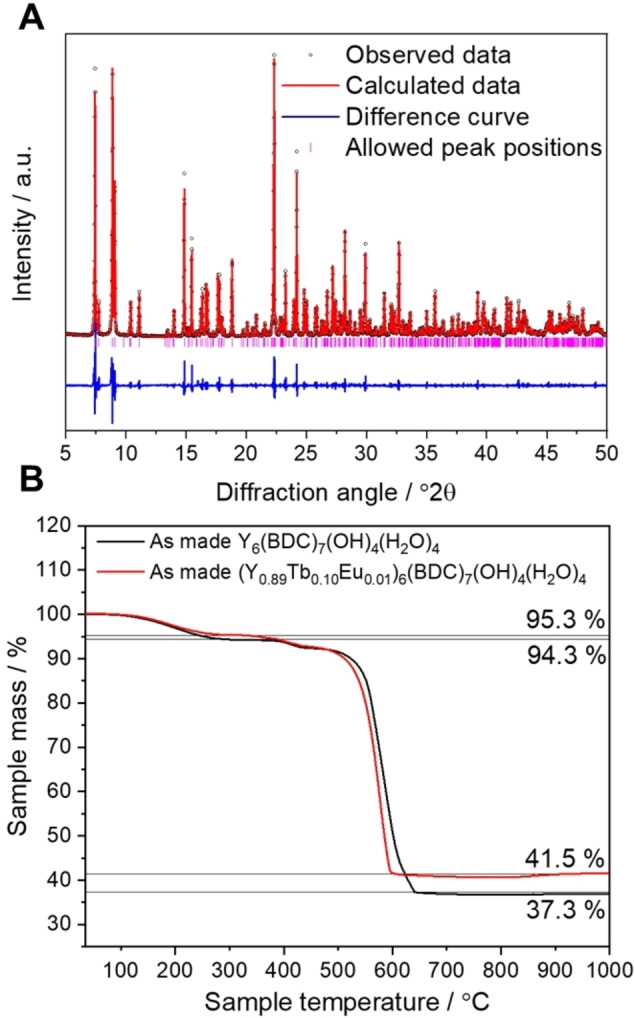
(A) Fitted powder XRD pattern of as made (Y_0.89_Tb_0.10_Eu_0.01_)_6_‐MOF with black circles representing the observed data points, red fitted profile, blue difference curve and pink tick marks representing allowed peaks (see Table 1 for refined lattice parameters). (B) Thermogravimetric analysis of as‐made (Y_0.89_Tb_0.10_Eu_0.01_)_6_‐MOF (red) compared to that of as‐made pure Y_6_‐MOF (black).

Confirmation of the successful synthesis was obtained via a combination of powder X‐ray diffraction (Figure 2A) and thermogravimetric analysis (TGA) (Figure 2B). The thermal decomposition of the parent Y_6_‐MOF is characterised by three steps; solvent loss, loss of bound water molecules and finally linker combustion to leave the mixed‐rare‐earth oxide. The decomposition of the substituted material follows that of Y_6_‐MOF and both traces are consistent with their theoretical mass losses. The refined unit cell parameters of simulated Y_6_‐MOF and the as‐made (Y_0.89_Tb_0.10_Eu_0.01_)_6_ MOF, Table 1, show an expansion of the unit cell for the substituted material. This expansion is consistent with the incorporation of the larger Eu and Tb cations into the Y‐based material. The concentration of lanthanides within the as made (Y_0.89_Tb_0.10_Eu_0.01_)_6_‐MOF was then measured by X‐ray fluorescence spectroscopy, showing close agreement with the intended substituent concentrations with an Y : Tb : Eu ratio of 89.2 : 9.6 : 1.2.

**Table 1:** Refined lattice parameters of as made (Y_0.89_Tb_0.10_Eu_0.01_)_6_‐MOF compared to those of the published structure of Y_6_‐MOF (space group *P* $\bar{1}$ ).^[20]^

Lattice Parameter	Y_6_‐MOF	(Y_0.89_Tb_0.10_Eu_0.01_)_6_‐MOF
a/Å	11.3918(11)	11.4091(25)
b/Å	12.0698(12)	12.0906(9)
c/Å	12.9957(13)	13.0132(26)
α (°)	86.8980(10)	86.906(5)
β (°)	67.1760(10)	67.130(5)
γ (°)	72.2000(10)	72.1862(21)
V/Å^3^	1563.9(3)	1570.34(21)

The thermal stability of (Y_0.89_Tb_0.10_Eu_0.01_)_6_‐MOF was investigated by *in situ* XRD upon heating (Figure S2), where it was determined that the MOF was stable up to around 400 °C before crystallinity was lost. The long‐term stability was further investigated by cycling the MOF between room temperature and 300 °C twice. Comparing powder XRD patterns before and after the heat cycling showed no significant changes indicating that the MOF was stable on repeated heating to 300 °C (Figure S3).

**Figure 3:**
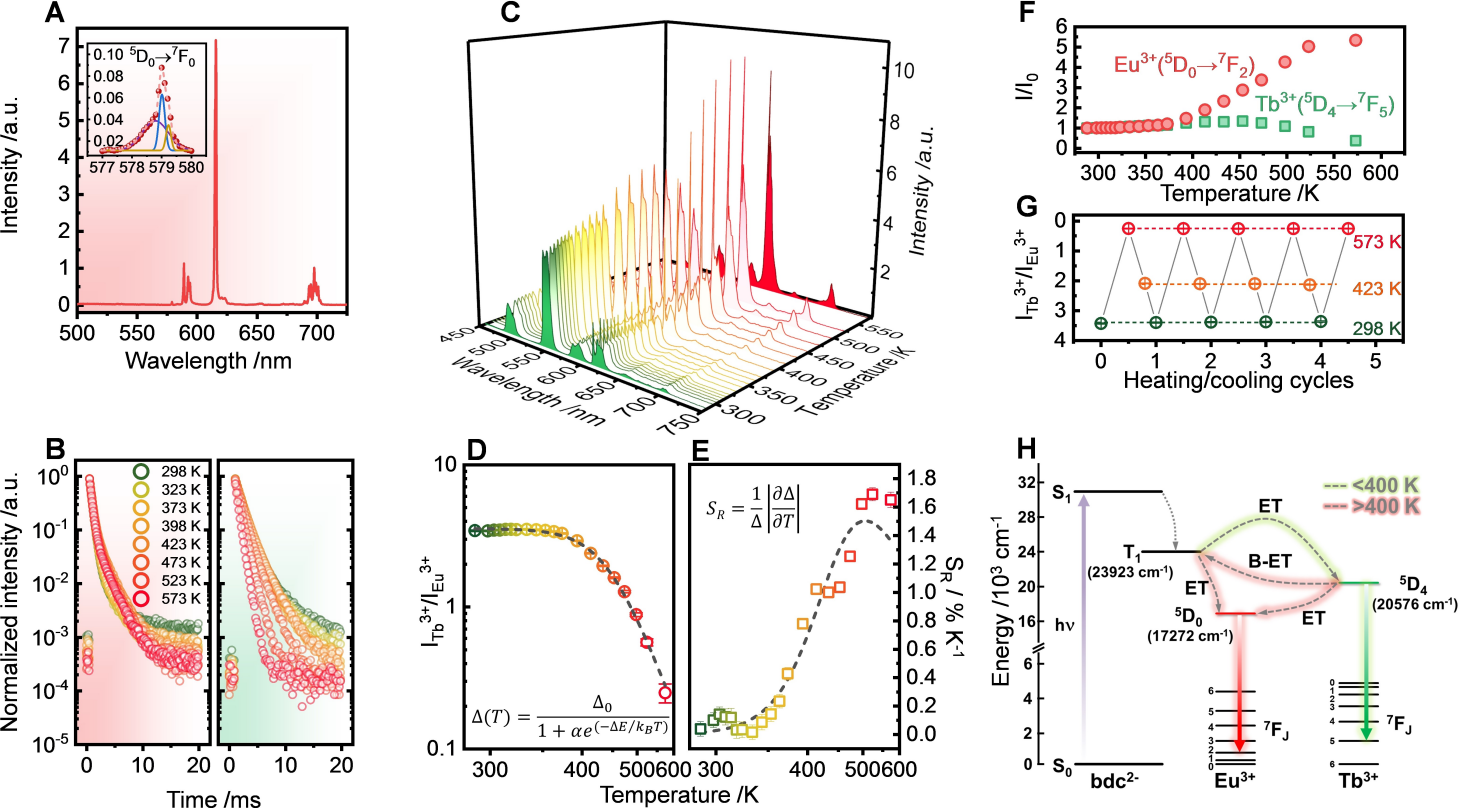
(A) Emission spectrum (λ_exc_=325 nm) of the (Y_0.99_Eu_0.01_)_6_‐MOF system; the inset shows an amplification of the ^5^D_0_→^7^F_0_ emission, which was deconvoluted into three Gaussian components. (B) Temperature dependence of the luminescence decays of (Y_0.99_Eu_0.01_)_6_‐MOF (left, red background, ^5^D_0_ level) and (Y_0.9_Tb_0.10_)_6_‐MOF (right, green background) under λ_exc_=325 nm excitation. (C) Temperature‐dependent emission spectra (λ_exc_=325 nm, 288–573 K) of (Y_0.89_Tb_0.10_Eu_0.01_)_6_‐MOF after thermal activation (573 K, 3 h). Thermal dependence of (D) the thermometric parameter Δ=$I_{{Tb}^{3+}}$ /$I_{{Eu}^{3+}}$ and (E) the relative thermal sensitivity (S_R_); the black dotted lines correspond to modelled data using Equations (1) and (2). (F) Integrated intensities of the ^5^D_4_→^7^F_5_ (544 nm, green squares) and ^5^D_0_→^7^F_2_ (610 nm, red circles) transitions normalized to their corresponding values at 288 K (I_0_). (G) Repeatability test for the thermometric parameter Δ=$I_{{Tb}^{3+}}$ /$I_{{Eu}^{3+}}$ via temperature cycling between 298 and 573 K. (H) Energy level diagram showing the energy transfer mechanisms in the (Y_0.89_Tb_0.10_Eu_0.01_)_6_‐MOF system (abbreviations: S, singlet; T, triplet; hν, energy absorption; ET, energy transfer, B‐ET, back‐energy transfer).

The BDC ligand is a known antenna chromophore for sensitising lanthanoid ions, especially Tb^3+^ and Eu^3+^.[^24^, ^25^, ^26^] Under 325 nm excitation, the as‐prepared MOF displayed the typical green and red luminescence of Tb^3+^ and Eu^3+^ ions (Figure S4). (Y_0.9_Tb_0.10_)_6_‐MOF exhibited emissions at 486, 544, 587, 621, 650, 668, and 680 nm attributed to the ^5^D_4_→^7^F_6‐0_ transitions of Tb^3+^, and (Y_0.99_Eu_0.01_)_6_‐MOF presented the characteristic set of Eu^3+ 5^D_0_→^7^F_0‐4_ signals at 579, 592, 615, 653 and 698 nm. The mixed (Y_0.89_Tb_0.10_Eu_0.01_)_6_‐MOF showed the characteristic Tb^3+^ and Eu^3+^ emissions simultaneously (Figure S5) under ligand excitation. No detectable luminescence from the BDC ligand could be observed in these compounds, attesting an efficient energy transfer process to the excited state of the Ln^3+^ ions. This sensitisation is further confirmed by the overall quantum yields (Table S2), which were 26, 7, and 17 % for (Y_0.9_Tb_0.10_)_6_‐MOF, (Y_0.99_Eu_0.01_)_6_‐MOF, and (Y_0.89_Tb_0.10_Eu_0.01_)_6_‐MOF respectively. These values are similar to those reported for Tb_2_(BDC)_3_(H_2_O)_4_ and Eu_2_(BDC)_3_(H_2_O)_4_.^[24]^ The emission spectrum of the (Y_0.99_Eu_0.01_)_6_‐MOF at 77 K (Figure 3A) also confirmed that the trivalent cations occupy multiple non‐equivalent sites of low symmetry in the triclinic *P* $\bar{1}$ structure. The broad and asymmetric ^5^D_0_→^7^F_0_ signal could be deconvoluted in three Gaussian components centred at 17265, 17271, and 17279 cm^−1^, in agreement with the three cation sites in the asymmetric unit of the hexanuclear cluster (Figure 1).

The temperature dependence of Tb^3+^ (^5^D_4_) and Eu^3+^ (^5^D_0_) excited state dynamics for (Y_0.9_Tb_0.10_)_6_‐MOF and (Y_0.99_Eu_0.01_)_6_‐MOF was investigated via decay curves (Figures 3B and S6). Owing to the occupation of multiple sites, the decays were fitted well with biexponential decay functions, with ^5^D_4_ decays showing a pseudo‐first order behaviour at higher temperatures. The ^5^D_0_ lifetimes in (Y_0.99_Eu_0.01_)_6_‐MOF remained approximately constant in the whole evaluated temperature range, except for a slight increase at about 398 K due to the elimination of water molecules from the Eu^3+^ coordination sphere.^[24]^ By contrast, the ^5^D_4_ lifetime of (Y_0.9_Tb_0.10_)_6_‐MOF decreased substantially when the temperature was increased above 423 K. This behaviour is attributed to a thermally activated non‐radiative energy transfer mechanism within the energy levels of the emitting centres and the ligand.[^27^, ^28^] Lowering the Tb^3+^ substitution concentration in (Y_0.9_Tb_0.10_)_6_‐MOF allowed for the detection of the ligand‐centered emissions bands (Figure S7). Time‐resolved phosphorescence spectra recorded at 77 K confirmed a triplet state energy (T_1_) around 23923 cm^−1^ for the BDC ligand, in agreement with previously reported values.^[26]^ Despite the efficient energy transfer from BDC to the acceptor levels of the Ln^3+^ ions (Figure S8), the energy gap between the T_1_ level and the ^5^D_0_ emitting state of Eu^3+^ (6651 cm^−1^) is much bigger compared to the difference between T_1_ and the ^5^D_4_ of Tb^3+^ (3347 cm^−1^). Hence, thermally driven depopulation of the ^5^D_4_ state by back‐transfer from Tb^3+^ to BDC is much more likely to occur than back‐transfer from the ^5^D_0_ level of Eu^3+^ to the ligand.

The energy level scheme of (Y_0.99_Eu_0.01_)_6_‐MOF and (Y_0.9_Tb_0.10_)_6_‐MOF suggested that the doubly‐substituted (Y_0.89_Tb_0.10_Eu_0.01_)_6_‐MOF could provide a high‐sensitivity ratiometric thermal response based on luminescence intensities. However, the temperature‐dependent luminescence spectra of (Y_0.89_Tb_0.10_Eu_0.01_)_6_‐MOF recorded between 77 and 300 K showed both Tb^3+^ and Eu^3+^ emissions remained practically unchanged at this low temperature range (Figure S9). Such behaviour contrasts with the common observation concerning the Eu^3+^/Tb^3+^ couple on Ln organic frameworks, where a decrease in Tb^3+^ luminescence is generally followed by a simultaneous increase in Eu^3+^ emissions in this temperature range.[^18^, ^20^, ^29^, ^30^, ^31^, ^32^, ^33^] This difference is tentatively explained by a stable and highly efficient ligand‐to‐Ln^3+^ energy transfer, which was proposed by Zhao et al. for [(Tb,Eu)(bpda)(NO_3_)(DMF)_2_](DMF) MOFs (bpda=biphenyl‐3,5‐dicarboxylate), in which Ln^3+^ emissions also remained practically constant between 10 and 300 K.^[34]^

The unusual luminescent behaviour presented by (Y_0.89_Tb_0.10_Eu_0.01_)_6_‐MOF with its high thermal stability enabled this mixed‐metal MOF to operate as a ratiometric luminescent thermometer at temperatures higher than usually explored for lanthanoid MOFs, i.e., up to 573 K. We initially evaluated the stability of UV‐excited luminescence against partial dehydration caused by a thermal activation process at 573 K for 3 h (Figure S10). The activated (Y_0.89_Tb_0.10_Eu_0.01_)_6_‐MOF displayed an additional signal at 610 nm when compared to the as‐prepared sample. This new signal arose between 373 and 398 K and it is attributed to a crystal‐field component of the ^5^D_0_→^7^F_2_ transition of Eu^3+^, which is hypersensitive towards changes in the chemical environment.^[35]^ Daiguebonne et al. reported a similar behaviour for the dehydration of Eu_2_(BDC)_3_(H_2_O)_4_.^[24]^ No additional alteration was verified after a second heating cycle at 573 K, thus excluding structural changes induced by heating. Powder XRD showed that crystallinity is maintained on cycles of dehydration‐rehydration (Figure S3). Interestingly, the hydration‐dehydration process is also reversible in terms of luminescence profiles (Figure S10c), suggesting that solid (Y_0.89_Tb_0.10_Eu_0.01_)_6_‐MOF could be further explored as a ratiometric sensor for water vapour. Considering the spectral alteration induced by the activation process, all subsequent measurements for (Y_0.89_Tb_0.10_Eu_0.01_)_6_‐MOF were conducted for powders previously activated at 573 K.

The temperature dependence of UV‐excited luminescence from 288 to 573 K for powders (Figure 3C−G and Figure S11a) and from 283 to 343 K for aqueous suspensions (Figure S12a) demonstrated the thermometric potential of (Y_0.89_Tb_0.10_Eu_0.01_)_6_‐MOF. Even though the intensities of ^5^D_4_→^7^F_5_ (544 nm, Tb^3+^) and ^5^D_0_→^7^F_2_ (610 nm, Eu^3+^) transitions remained practically unchanged between 288–373 K, further temperature increase intensified the emissions of Eu^3+^, whilst Tb^3+^ intensities decreased (Figure 3C and 3F). Such a dramatic intensity change resulted in a marked alteration of the emission colour, which was systematically tuned from green (288 K), to orange (498 K), to red (573 K). Thus, temperature can be qualitatively assessed by the naked eye, implying that (Y_0.89_Tb_0.10_Eu_0.01_)_6_‐MOF powder acts as a visual thermosensor under UV excitation (Figure S11b). For water suspensions, transition intensities of both Ln^3+^ were gradually reduced as the temperature increased (Figure S12a,b), indicating the presence of non‐radiative decay pathways associated with the interaction of the solids with the solvent. This effect was more pronounced for Eu^3+^ emissions as the integrated intensity decrease (51 %) was higher than that for Tb^3+^ (37 %) due to the hypersensitivity of the ^5^D_0_→^7^F_2_ transition.^[35]^

The thermometric parameter Δ=$I_{{Tb}^{3+}}$ /$I_{{Eu}^{3+}}$ based on the intensity ratio between the main emissions of Tb^3+^ and Eu^3+^ was used to quantify the thermal sensing ability of (Y_0.89_Tb_0.10_Eu_0.01_)_6_‐MOF (Eq. S1). The temperature dependence of the thermometric parameter Δ in the 288–573 K range is presented in Figure 3D, which was described in terms of the classical Mott‐Seitz model[^36^, ^37^] for a single non‐radiative recombination channel, according to Equation 1:^[38]^

(1) $$\Delta \left(T\right)=\;\frac{\Delta_{0}}{1+\alpha e^{-\Delta E/k_{B}T}}$$

where Δ_0_ is the Δ parameter when T→0 K, α is the ratio between the non‐radiative (at T→0 K) and the radiative rates, and ΔE is the activation energy for the non‐radiative channel. The black dotted line in Figure 3D is the temperature calibration curve for the Δ parameter, which yielded Δ_0_=3.54±0.03, α=(8.62±0.48)×10^4^, and ΔE=3518±241 cm^−1^, with r^2^>0.996. This non‐radiative channel is consistent with the thermally driven depopulation of the ^5^D_4_ state of Tb^3+^ through the ligand levels via metal‐to‐ligand back energy transfer (B‐ET), as the energy gap between the T_1_ state and ^5^D_4_ level (3347 cm^−1^, Figure S8) is statistically equal to the activation energy estimated from this model (Figure 3H). Conversely, a linear profile was observed for the Δ parameter for the (Y_0.89_Tb_0.10_Eu_0.01_)_6_‐MOF in suspension between 283 and 343 K (Figure S12c). The intensity ratio profiles confirmed the versatility of the proposed system, which can be used as a ratiometric luminescent thermometer both as a powder (288–573 K) and as an aqueous suspension (283–343 K).

The thermometric performance of the (Y_0.89_Tb_0.10_Eu_0.01_)_6_‐MOF was quantified by the relative thermal sensitivity (S_R_, % K^−1^), which indicates the percentual change of the Δ parameter per unit of temperature change (Eq. (2)) and enables comparison between different thermometers regardless of their nature:^[38]^

(2) $$S_{R}=\;\frac{1}{\Delta }\left|\frac{\partial \Delta }{\partial T}\right|\;$$

The relative sensitivity of (Y_0.89_Tb_0.10_Eu_0.01_)_6_‐MOF as both powder and water suspension was practically constant between 288 and 353 K, with average values about 0.10±0.05 % K^−1^ (solid, Figure 3E) and 0.35±0.15 % K^−1^ (suspension, Figure S13). The higher sensitivity in the aqueous suspension in this temperature range is due to the large intensity variation of the ^5^D_0_→^7^F_2_ Eu^3+^ transition, as discussed previously. These values are similar to those reported by Cadiau et al. for (Tb,Eu)_2_(BDC)_3_(H_2_O)_4_ (0.31 % K^−1^ and 0.14 % K^−1^ at 318 K for suspension and solid, respectively),^[25]^ but were considerably lower than those presented by Pan et al.^[30]^ for [(CH_3_)_2_NH_2_](Eu,Tb)(bptc) (9.42 % K^−1^ at 310 K, bptc=biphenyl‐3,3’,5,5’‐tetracarboxylic acid). However, relative sensitivities as low as 0.2 % K^−1^ are adequate for thermometry in biological systems,[^39^, ^40^, ^41^] indicating the potential applicability of the prepared Ln‐MOFs for operation in the physiological temperature range (298–318 K), for example. The maximum relative thermal sensitivity achieved for the powder sample was 1.69±0.04 % K^−1^ at 523 K. This is the highest relative thermal sensitivity reported so far for Ln‐MOFs at such high temperatures, as shown by a comprehensive comparison between the results of this work and other reported ratiometric thermometers (Table S3).

The repeatability of the thermometer was measured over five consecutive temperature cycles between 298 and 573 K (Figure 3G and Figure S14a). Given the high thermal stability of (Y_0.89_Tb_0.10_Eu_0.01_)_6_‐MOF, the Δ parameter was fully reversible without significant hysteresis, with repeatability better than 98 %. Similar findings were achieved for water suspension (repeatability better than 97 %) after three heating‐cooling cycles in the 293–318 K range (Figure S14b).

The outstanding thermal and water stability of (Y_0.89_Tb_0.10_Eu_0.01_)_6_‐MOF enables a very broad operational temperature range for high sensitivity optical thermometry, which has never been reported so far for Ln‐MOF materials. In general, effective thermometry applications of Ln‐MOFs are limited to the cryogenic (<100 K–298 K) and biological (298–313 K) temperature ranges due to their low thermal stability.[^20^, ^33^, ^42^, ^43^] Few works report the potential application of Ln‐MOF as luminescent thermometers at higher temperatures, where maximum temperatures did not exceed 473 K.[^44^, ^45^, ^46^] In addition, the calculated temperature uncertainties also support the potentiality for (Y_0.89_Tb_0.10_Eu_0.01_)_6_‐MOF to accurately detect temperature fluctuations. The temperature uncertainty, δT (Eq. S2), indicates the smallest change in temperature that can theoretically be detected using the thermometric parameter^[38]^ Δ=$I_{{Tb}^{3+}}$ /$I_{{Eu}^{3+}}$ . For powders, the temperature uncertainties ranged from ∼0.2 to ∼0.01 K between 353 and 573 K; similar values of δT were found at 283–343 K for aqueous suspensions (Figure S15).

From a fundamental point of view, the thermometric performance of the (Y_0.89_Tb_0.10_Eu_0.01_)_6_‐MOF is described not only in terms of a back energy transfer process from Tb^3+^ to the ligands, but also of a Tb^3+^→Eu^3+^ energy transfer mechanism. This process could be discerned at 473 K by the presence of the ^5^D_4_←^7^F_6_ transition of Tb^3+^ (486 nm) in the excitation spectra of (Y_0.89_Tb_0.10_Eu_0.01_)_6_‐MOF when the ^5^D_0_→^7^F_4_ emission of Eu^3+^ (698 nm) was monitored (Figure S16). Conversely, the room temperature excitation spectrum monitoring the same Eu^3+^ emission showed no Tb^3+^ absorptions, thus indicating the extent of Tb^3+^→Eu^3+^ energy transfer is indeed dependent on the temperature. We further confirmed this behaviour measuring an emission spectrum of (Y_0.89_Tb_0.10_Eu_0.01_)_6_‐MOF under selective Tb^3+^ excitation at 486 nm (Figures S17 and S18). Whilst the Tb^3+^ luminescence became progressively lower with the increase in the temperature, the Eu^3+^ emissions gradually enhanced on the monitored range, even though the excitation energy was not high enough to directly populate the energy levels of the BDC ligands. Interestingly, comparing the thermometric responses provided for the Ln‐MOF under λ_exc_=486 nm (Tb^3+^ absorption) and λ_exc_=325 nm (BDC absorption), similar trends were found (Figure S17). The Δ parameter was also well fitted by Equation (1), yielding an activation energy for the non‐radiative channel (ΔE) about 3789±310 cm^−1^. This value is the same of that found under ligand excitation (ΔE=3518±241 cm^−1^) within the experimental error, which also corresponds to the energy difference between ligand T_1_ state and the Tb^3+ 5^D_4_ level. Hence, both Tb^3+^‐to‐Eu^3+^ phonon‐assisted energy transfer and Tb^3+^‐to‐ligand back‐transfer mechanisms are operative in this system, also enabling use of visible excitation (λ_exc_=486 nm) to attain thermometric correlations.

**Figure 4:**
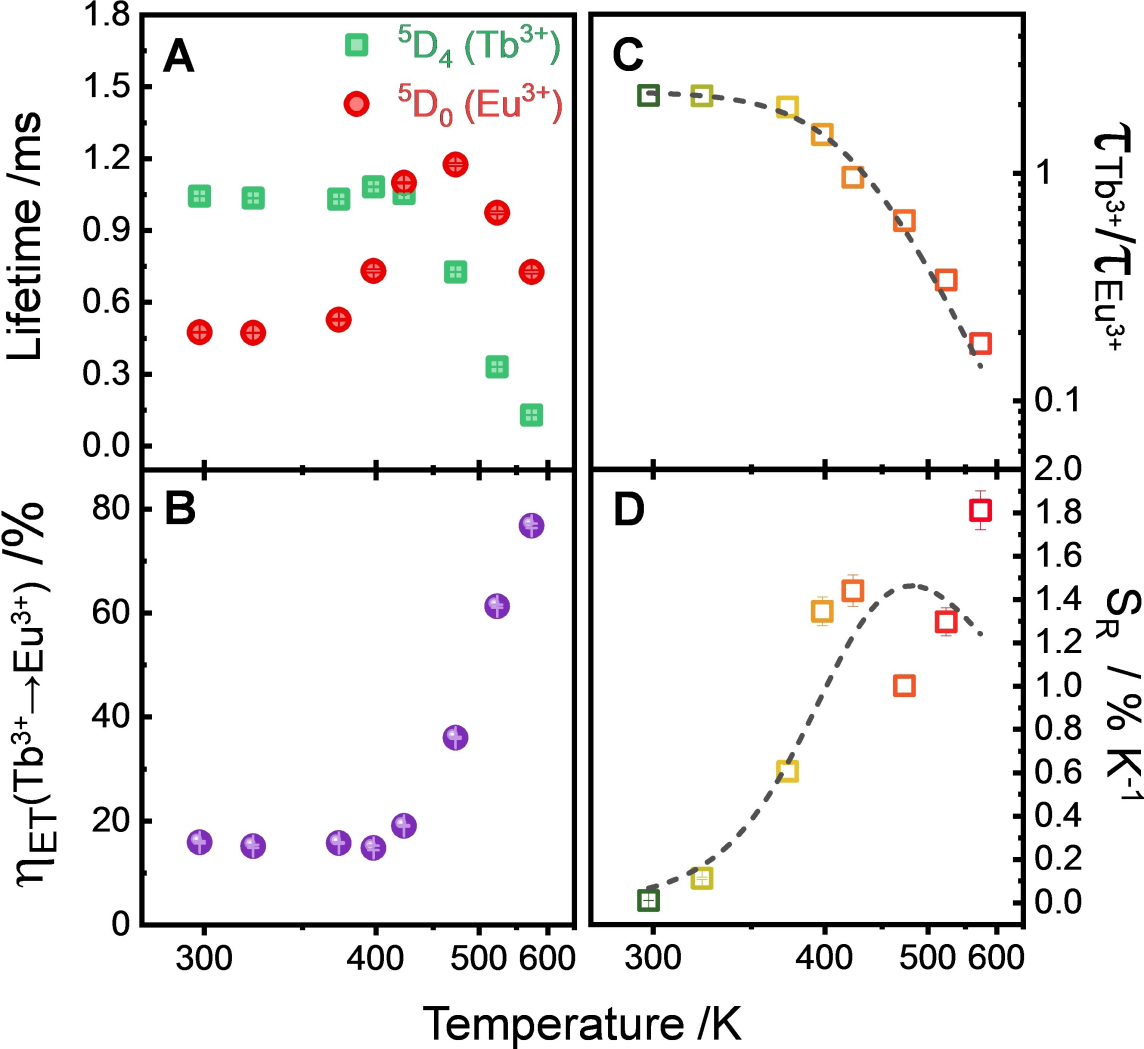
(A) Dependence of the ^5^D_4_ (τ_Tb_^3+^, green squares) and ^5^D_0_ (τ_Eu_^3+^, red circles) lifetimes against temperature from 298 to 573 K for (Y_0.89_Tb_0.10_Eu_0.01_)_6_‐MOF powder. Decay curves and lifetimes are shown in Figure S19 and Table S3. (B) Temperature dependence of the energy transfer efficiency (η_ET_) from Tb^3+^ to Eu^3+^ ions. (C) Variation of the ratio between Tb^3+^ and Eu^3+^ lifetimes (τ_Tb_^3+^/τ_Eu_^3+^) against the temperature, and (D) relative thermal sensitivity of (Y_0.89_Tb_0.10_Eu_0.01_)_6_‐MOF powder using the τ_Tb_^3+^/τ_Eu_^3+^ ratio as a thermometric parameter. Dotted lines in (C) and (D) correspond to fittings using Equations (1) and (2), respectively.

Decay profiles of ^5^D_4_ and ^5^D_0_ states monitoring the emissions at 544 nm (Tb^3+^, ^5^D_4_→^7^F_5_) and 698 nm (Eu^3+^, ^5^D_0_→^7^F_4_) (Figure S19) brought further evidence of energy transfer between Ln^3+^ emitting centres in the mixed (Y_0.89_Tb_0.10_Eu_0.01_)_6_‐MOF. Irrespective of the temperature evaluated, this system showed shorter ^5^D_4_ lifetimes in comparison to the singly‐substituted (Y_0.9_Tb_0.10_)_6_‐MOF. By contrast, the mixed (Y_0.89_Tb_0.10_Eu_0.01_)_6_‐MOF showed higher ^5^D_0_ lifetimes in comparison to (Y_0.99_Eu_0.01_)_6_‐MOF from 398 to 573 K (Figure S20 and Table S4). Also, observation of characteristic ^5^D_0_ rise times corroborated the coupling between ^5^D_4_ and ^5^D_0_ emitting states by energy transfer (Figure S21, Table S5). The time elapsed for the population of the ^5^D_0_ before the beginning of the emission decay was approximately constant for the singly‐ substituted (Y_0.99_Eu_0.01_)_6_‐MOF. However, these rise times were significantly longer for the mixed (Y_0.89_Tb_0.10_Eu_0.01_)_6_‐MOF at high temperatures, thus explaining why the decrease of the ^5^D_4_ lifetimes with temperature is associated with an increase of the ^5^D_0_ lifetimes from 398 K (Figure 4A).

The efficiency (η_ET_) of this energy transfer process was estimated from Equation 3:^[47]^

(3) $$\eta_{ET}=1-\frac{\tau }{\tau_{0}},\;$$

where τ_0_ and τ are the ^5^D_4_ lifetimes of the Tb^3+^ ions in (Y_0.9_Tb_0.10_)_6_‐MOF and (Y_0.89_Tb_0.10_Eu_0.01_)_6_‐MOF, respectively. As expected, the energy transfer efficiency substantially increases at temperatures higher than 398 K (Figure 4B) and reach the maximum about 77 % around 573 K. This is consistent with the enhancement of the Eu^3+^ emission at the expense of Tb^3+^ quenching, thus culminating in an increase of the relative thermal sensitivity over this temperature range. Such an energy transfer process is mainly governed by phonon‐assisted multipolar mechanism.[^47^, ^48^] Furthermore, the correlation between the Eu^3+^ and Tb^3+^ decay kinetics enable the use of luminescence lifetimes as an additional thermometric parameter for thermal sensing by the MOF system (Figures 4C and 4D). The ratio between the ^5^D_4_ and ^5^D_0_ lifetimes (i.e., τ_Tb_^3+^/τ_Eu_^3+^) followed the same profile of luminescence intensities, confirming the adequateness of the Mott‐Seitz model (Eq. (1)) for this system. The ΔE value calculated from lifetime dependence on temperature was ΔE=3010±360 cm^−1^, also in very good agreement with the determinations performed via luminescence intensities.

The luminescence of Eu^3+^ and Tb^3+^ in (Y_0.89_Tb_0.10_Eu_0.01_)_6_‐MOF is coupled by these energy transfer mechanisms, but non‐radiative effects induced by water molecules operate more prominently on the Eu^3+^ centres, as previously discussed. The high Brønsted acidity of μ_3_‐OH groups and the easily exchangeable terminal H_2_O molecules^[21]^ suggested the (Y_0.89_Tb_0.10_Eu_0.01_)_6_‐MOF may undergo a high degree of deuteration if exposed to heavy water (D_2_O). Hence, the well‐known consequences of OH/OD exchange in Ln complexes[^49^, ^50^] suggested us the (Y_0.89_Tb_0.10_Eu_0.01_)_6_‐MOF could not only afford thermometric response, but also act as a luminescent sensor for heavy water (D_2_O). Because of the lower energy gap between the emitting level and the ground state terms, the luminescence lifetime of Eu^3+^ is expected to be strongly dependent on the OH/OD exchange and hence of the molar fraction between H_2_O and D_2_O in suspension (X${}_{H{}_{2}O}$ ). Also, the ^5^D_0_ lifetimes are rather insensitive towards temperature changes at moderate temperatures (298–350 K), so Eu^3+^ decays can act as a stable probe for the H_2_O/D_2_O molar fraction in liquid phase.

**Figure 5:**
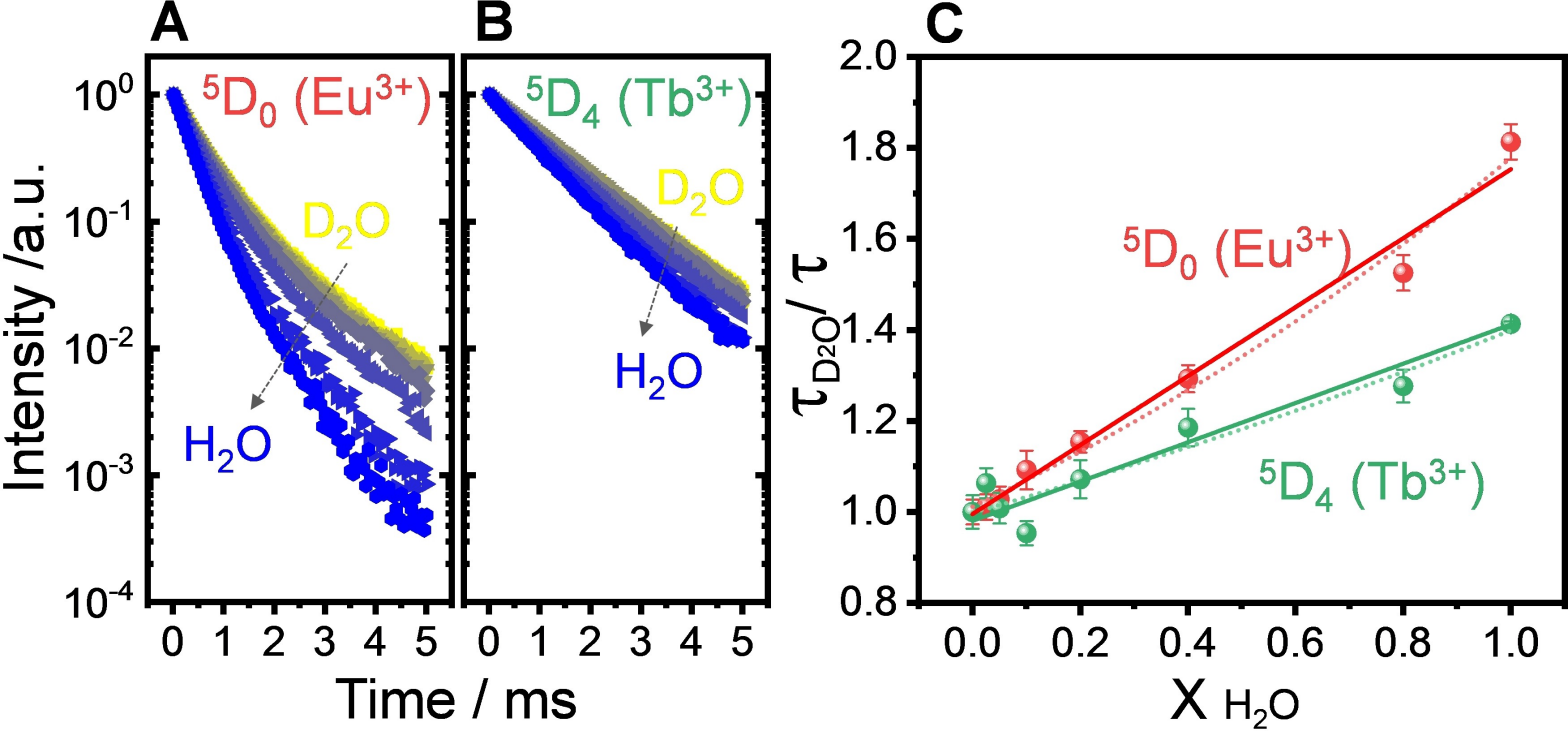
Effect of the H_2_O/D_2_O molar fraction (X${}_{H{}_{2}O}$ ) on the luminescence decay profiles of (Y,Tb,Eu)_6_‐MOF 3 mg mL^−1^ aqueous suspensions (λ_exc_=325 nm) at 298 K monitoring (A) the ^5^D_0_ level of Eu^3+^ (^5^D_0_→^7^F_4_, 698 nm) and (B) the ^5^D_4_ level of Tb^3+^ (^5^D_4_→^7^F_5_, 544 nm). Yellow points denote measurements in pure D_2_O and blue points correspond to measurements in pure H_2_O. (C) Stern‐Volmer‐type relationship between luminescence lifetimes of Eu^3+^ (^5^D_0_, red) and Tb^3+^ (^5^D_4_, green) and the H_2_O/D_2_O molar fraction. The vertical axis corresponds to the ratio between lifetimes in pure D_2_O (τ${}_{D{}_{2}O}$ ) and lifetimes (τ) at a given H_2_O/D_2_O molar fraction calculated from bi‐exponential fits of (A) and (B). Solid lines correspond to linear fits of the type y_0_/y=1+K_ap_x (Eu^3+^: r^2^=0.9836, K_ap_=0.7573; Tb^3+^: r^2^=0.9747, K_ap_=0.4314), and dotted lines correspond to exponential correlations of the type ln(y_0_/y)=K_ap_x (Eu^3+^: r^2^=0.9857, K_ap_=0.5643; Tb^3+^: r^2^=0.9163, K_ap_=0.3373).

We acquired ^5^D_0_ decay profiles of different 3 mg mL^−1^ aqueous suspensions of (Y_0.89_Tb_0.10_Eu_0.01_)_6_‐MOF in H_2_O/D_2_O at 298 K; pure H_2_O and D_2_O suspensions were mixed to attain different H_2_O/D_2_O molar fractions (Figure 5A and 5B). The increase in the X${}_{H{}_{2}O}$ fraction resulted on the decrease of the decay lifetimes due to the enhanced non‐radiative contribution. Given the larger energy gap between the ^5^D_4_ and the ^7^F_J_ states, Tb^3+^ lifetimes afforded a lower sensitivity towards OH/OD exchange (Figure 5B). The Eu^3+ 5^D_0_ luminescence lifetimes obtained from bi‐exponential fits of experimental decays obeyed a Stern‐Volmer‐type relation with respect to the H_2_O/D_2_O fraction (Figure 5C), thus enabling optical determination of the D_2_O content in H_2_O. Assuming a classical linear Stern‐Volmer relation (y_0_/y=1+K_ap_x, where K_ap_ is an apparent Stern‐Volmer constant), a correlation coefficient of r^2^=0.9836 was obtained for the Eu^3+^ decays, with a slope of 0.7573 with respect to the X${}_{H{}_{2}O}$ fraction. This result denotes a lower sensitivity in comparison to recently reported luminescent D_2_O sensors,^[51]^ but the general behaviour confirms the effectiveness of the MOF towards D_2_O detection in the full compositional range. Assuming an exponential Stern‐Volmer‐type relation (ln(y_0_/y)=K_ap_x, i.e., a positive deviation on y_0_/y due to large extent of quenching by OH oscillators), a higher correlation coefficient is obtained (r^2^=0.9857), with a slope of 0.5643. Given the labile nature of RE‐based coordination compounds, the OH/OD exchange process is fast (i.e., <2 min until unchanged intensity vs. time profiles), and the lifetime response of the (Y,Tb,Eu)_6_‐MOF towards H_2_O or D_2_O exposure is shown to be reversible within ±2.5 % under alternated exposure to X${}_{H{}_{2}O}$ =0.8 and X${}_{H{}_{2}O}$ =0.2 conditions (Figure S22). Because of the limited number of D_2_O detection materials and the importance of this kind of sensing for chemical analysis and nuclear power production,^[51]^ the results obtained herein confirm this additional potentiality of the (Y_0.89_Tb_0.10_Eu_0.01_)_6_‐MOF material.

## Conclusion

We have demonstrated the applicability of (Y_0.89_Tb_0.10_Eu_0.01_)_6_‐MOF in ratiometric luminescence thermometry, and in quantitative sensing of D_2_O in H_2_O. Because of its outstanding thermal and water stability, the material displays excellent thermometric correlations both as aqueous suspension (283–343 K) and in the solid‐state (288–573 K), with maximum relative thermal sensitivities of 0.35±0.15 % K^−1^ and 1.69±0.04 % K^−1^ at 523 K, respectively. Both Tb^3+^‐to‐Eu^3+^ phonon‐assisted energy transfer and Tb^3+^‐to‐ligand back transfer mechanisms govern excited state dynamics of (Y_0.89_Tb_0.10_Eu_0.01_)_6_‐MOF, being responsible for the different temperature‐dependent luminescence of Tb^3+^ and Eu^3+^. This is the first report of a Ln‐based MOF acting as a ratiometric luminescent thermometer in the 288–573 K temperature range, which opens new perspectives for non‐contact thermometry and multifunctional sensing. New perspectives can be envisioned, as the design of a multiplatform for *in operando* temperature sensing of catalytic reactions.[^9^, ^52^, ^53^] Indeed, one of the many important applications of MOFs concerns the application of their intrinsic porosity and Lewis acidity for catalysis[^22^, ^54^] and the Yb‐analogue of the MOF we have studied here has already been proven as a solid‐acid catalyst.^[22]^ The system proposed herein points towards designing multifunctional materials that potentially can locally access temperature and enhance the rate of reactions of interest simultaneously, as well as probing the solvent environment. Our work should inspire further experimental studies in this direction, as well as computational efforts to rationalise the observed properties.

## Experimental Section

The synthesis of (Y_0.89_Tb_0.10_Eu_0.01_)_6_(BDC)_7_(OH)_4_(H_2_O)_4_ was carried out using a hydrothermal method_._ Yttrium chloride hexahydrate (1.7 g, 5.61 mmol, Merck), europium chloride hexahydrate (23.1 mg, 0.06 mmol, Strem Chemicals) and terbium chloride hexahydrate (235 mg, 0.63 mmol, Alpha Aesar) were added to a 100 mL PTFE autoclave liner. To this, disodium terephthalate (1.575 g, 7.5 mmol, Alpha Aesar) was added followed by 50 mL deionised water and finally 2.2 g of 2 M aqueous sodium hydroxide solution. The synthesis mixture was stirred for 5 min and then sealed and heated to 190 °C for 72 h. After cooling, the powder was collected by vacuum filtration and washed twice with water and twice with methanol before being left for a short time to dry in air. The powder was then returned to a 100 mL PTFE lined autoclave along with 50 mL deionised water and heated to 190 °C for a further 72 h. After cooling, the powder was recovered by vacuum filtration followed by washing twice with water and twice with methanol. The final product was dried at 70 °C in air to leave a highly crystalline white powder. The singly substituted materials were synthesised by the same method by addition of the desired molar ratios of each lanthanide precursor into the initial reaction mixture.

Powder XRD patterns were measured at room temperature using a Panalytical Empyrean diffractometer operating with Cu Kα_1/2_ radiation. The diffraction profile was fitted using the GSAS suite of software^1^ to obtained lattice parameters via the Pawley method. Non‐ambient powder XRD measurements were made using a Bruker D8 Advance diffractometer equipped with Cu Kα_1/2_ radiation and a VÅNTEC‐1 high‐speed detector. Powders were heated *in situ* using an Anton Paar XRK 900 reaction chamber controlled in air through a TCU 750 temperature unit. XRF spectra were acquired using a Rigaku Primus IV wavelength dispersive X‐ray fluorescence spectrometer equipped with a 4 kW X‐ray tube. Samples were scanned for approximately 5 min to obtain sufficient counts for quantitative analysis to be carried out.

Luminescence spectra were collected on a Fluorolog 3 (Horiba FL3‐22‐iHR320) spectrofluorometer equipped with double‐grating (1200 g mm^−1^) monochromators blazed at 330 nm (excitation) and 500 nm (emission). A Hamamatsu R928P photomultiplier tube was employed as a detector, and a 450 W xenon arc lamp was used as a radiation source. Luminescence spectra were corrected via software with respect to lamp intensity, optical response, and detector sensitivity. The temperature‐dependent spectra of the Ln_6_‐MOFs as powders were obtained between 77 and 573 K using a Linkam Scientific temperature‐controlled stage (THMS600). Excitation and emission signals were collected by optical fibres (Wavelength Electronics LFI‐3751). Spectra of water suspensions (1 mg mL^−1^) of Ln_6_‐MOFs were measured in quartz cuvettes (1 cm optical path, PTFE stopper, 3 mL) placed on a F3004 Peltier‐controlled sample holder (Horiba), with temperatures set from 288 to 343 K. Samples were thermalized (±0.1 K) for ∼10 min before each measurement at the selected temperatures. The time‐resolved phosphorescence spectra were collected at 77 K applying different time delays for the detection (10^−3^, 10^−2^, and 10^−1^ ms). Emission decay curves were collected on the same instrument using a TCSPC system and a 150 W xenon pulsed lamp as excitation source.

## Conflict of interest

The authors declare no conflict of interest.

1

## Supporting information

As a service to our authors and readers, this journal provides supporting information supplied by the authors. Such materials are peer reviewed and may be re‐organized for online delivery, but are not copy‐edited or typeset. Technical support issues arising from supporting information (other than missing files) should be addressed to the authors.

Supporting Information

## Data Availability

The data that support the findings of this study are available in the supplementary material of this article.

## References

[1] P. R. N. Childs , J. R. Greenwood , C. A. Long , Rev. Sci. Instrum. 2000, 71, 2959–2978.

[2] M. Toda , N. Inomata , T. Ono , I. Voiculescu , IEEJ Trans. Electr. Electron. 2017, 12, 153–160.

[3] R. Wu , H. G. Ge , C. F. Liu , S. H. Zhang , L. Hao , Q. Zhang , J. Song , G. H. Tian , J. G. Lv , Talanta 2019, 196, 191–196. 30683350 10.1016/j.talanta.2018.12.020

[4] C. Gota , K. Okabe , T. Funatsu , Y. Harada , S. Uchiyama , J. Am. Chem. Soc. 2009, 131, 2766–2767. 19199610 10.1021/ja807714j

[5] C. D. S. Brites , P. P. Lima , N. J. O. Silva , A. Millan , V. S. Amaral , F. Palacio , L. D. Carlos , New J. Chem. 2011, 35, 1177–1183.

[6] D. Jaque , F. Vetrone , Nanoscale 2012, 4, 4301–4326. 22751683 10.1039/c2nr30764b

[7] C. D. S. Brites , P. P. Lima , N. J. O. Silva , A. Millan , V. S. Amaral , F. Palacio , L. D. Carlos , Nanoscale 2012, 4, 4799–4829. 22763389 10.1039/c2nr30663h

[8] J. Lee , N. A. Kotov , Nano Today 2007, 2, 48–51.

[9a] C. Krishnaraj , H. Rijckaert , H. Sekhar Jena , P. Van Der Voort , A. M. Kaczmarek , ACS Appl. Mater. Interfaces 2021, 13, 47010–47018; 34570479 10.1021/acsami.1c11314

[9b] H. Sekhar Jena , H. Rijckaert , C. Krishnaraj , I. Van Driessche , P. Van Der Voort , A. M. Kaczmarek , Chem. Mater. 2021, 33, 8007–8017;

[9c] A. M. Kaczmarek , H. Sekhar Jena , C. Krishnaraj , H. Rijckaert , S. K. P. Veerapandian , A. Meijerink , P. Van Der Voort , Angew. Chem. Int. Ed. 2021, 133, 3771–3780. 10.1002/anie.202013377 33170988

[10] X. D. Wang , O. S. Wolfbeis , R. J. Meier , Chem. Soc. Rev. 2013, 42, 7834–7869. 23793774 10.1039/c3cs60102a

[11] S. Chorazy , M. Wyczesany , B. Sieklucka , Mol. 2017, 22, 30. 10.3390/molecules22111902 PMC6150171 29113065

[12] R. Nagaishi , T. Kimura , S. P. Sinha , Mol. Phys. 2003, 101, 1007–1014.

[13] S. Lis , J. Alloys Compd. 2002, 341, 45–50.

[14] G. R. Choppin , D. R. Peterman , Coord. Chem. Rev. 1998, 174, 283–299.

[15] J. V. Beitz , J. Alloys Compd. 1994, 207, 41–50.

[16] N. Sabbatini , M. Guardigli , J. M. Lehn , Coord. Chem. Rev. 1993, 123, 201–228.

[17] Y. J. Cui , R. J. Song , J. C. Yu , M. Liu , Z. Q. Wang , C. D. Wu , Y. Yang , Z. Y. Wang , B. L. Chen , G. D. Qian , Adv. Mater. 2015, 27, 1420–1425. 25581401 10.1002/adma.201404700

[18] Y. J. Cui , H. Xu , Y. F. Yue , Z. Y. Guo , J. C. Yu , Z. X. Chen , J. K. Gao , Y. Yang , G. D. Qian , B. L. Chen , J. Am. Chem. Soc. 2012, 134, 3979–3982. 22352469 10.1021/ja2108036

[19] H. Z. Wang , D. Zhao , Y. J. Cui , Y. Yang , G. D. Qian , J. Solid State Chem. 2017, 246, 341–345.

[20] Y. J. Cui , F. L. Zhu , B. L. Chen , G. D. Qian , Chem. Commun. 2015, 51, 7420–7431. 10.1039/c5cc00718f 25715078

[21] D. F. Weng , X. J. Zheng , L. P. Jin , Eur. J. Inorg. Chem. 2006, 4184–4190.

[22] D. L. Burnett , R. Oozeerally , R. Pertiwi , T. W. Chamberlain , N. Cherkasov , G. J. Clarkson , Y. K. Krisnandi , V. Degirmenci , R. I. Walton , Chem. Commun. 2019, 55, 11446–11449. 10.1039/c9cc05364f 31486470

[23] M. I. Breeze , T. W. Chamberlain , G. J. Clarkson , R. P. de Camargo , Y. Wu , J. F. de Lima , F. Millange , O. A. Serra , D. O′Hare , R. I. Walton , CrystEngComm 2017, 19, 2424–2433.

[24] C. Daiguebonne , N. Kerbellec , O. Guillou , J. C. Bunzli , F. Gumy , L. Catala , T. Mallah , N. Audebrand , Y. Gerault , K. Bernot , G. Calvez , Inorg. Chem. 2008, 47, 3700–3708. 18366158 10.1021/ic702325m

[25] A. Cadiau , C. D. S. Brites , P. Costa , R. A. S. Ferreira , J. Rocha , L. D. Carlos , ACS Nano 2013, 7, 7213–7218. 23869817 10.1021/nn402608w

[26] K. Panyarat , A. Ngamjarurojana , A. Rujiwatra , J. Photochem. Photobiol. A 2019, 377, 167–172.

[27] C. D. S. Brites , P. P. Lima , N. J. O. Silva , A. Millan , V. S. Amaral , F. Palacio , L. D. Carlos , Adv. Mater. 2010, 22, 4499–4504. 20803765 10.1002/adma.201001780

[28] Z. P. Wang , D. Ananias , A. Carne-Sanchez , C. D. S. Brites , I. Imaz , D. Maspoch , J. Rocha , L. D. Carlos , Adv. Funct. Mater. 2015, 25, 2824–2830.

[29] J. Q. Liu , L. Pei , Z. G. Xia , Y. Xu , Cryst. Growth Des. 2019, 19, 6586–6591.

[30] Y. Pan , H. Q. Su , E. L. Zhou , H. Z. Yin , K. Z. Shao , Z. M. Su , Dalton Trans. 2019, 48, 3723–3729. 30806435 10.1039/c9dt00217k

[31] D. A. Zhao , D. Yue , L. Zhang , K. Jiang , G. D. Qian , Inorg. Chem. 2018, 57, 12596–12602. 30256099 10.1021/acs.inorgchem.8b01746

[32] X. T. Rao , T. Song , J. K. Gao , Y. J. Cui , Y. Yang , C. D. Wu , B. L. Chen , G. D. Qian , J. Am. Chem. Soc. 2013, 135, 15559–15564. 24063306 10.1021/ja407219k

[33] J. Rocha , C. D. S. Brites , L. D. Carlos , Chem. Eur. J. 2016, 22, 14782–14795. 27482637 10.1002/chem.201600860

[34] D. Zhao , X. T. Rao , J. C. Yu , Y. J. Cui , Y. Yang , G. D. Qian , Inorg. Chem. 2015, 54, 11193–11199. 26575207 10.1021/acs.inorgchem.5b01623

[35] K. Binnemans , Coord. Chem. Rev. 2015, 295, 1–45.

[36] N. F. Mott , Proc. R. Soc. London A 1938, 167, 384–391.

[37] F. Seitz , Trans. Faraday Soc. 1939, 35, 74–85.

[38] C. D. S. Brites, A. Millán, L. D. Carlos, *A Handbook on the Physics and Chemistry of Rare Earths*, eds. P. J. C. G. and E. V. K., Elsevier, Amsterdam, **2016**, *49*, 339–427.

[39] B. del Rosal , E. Ximendes , U. Rocha , D. Jaque , Adv. Opt. Mater. 2017, 5.

[40] L. Labrador-Paez , M. Pedroni , A. Speghini , J. Garcia-Sole , P. Haro-Gonzalez , D. Jaque , Nanoscale 2018, 10, 22319–22328. 30468230 10.1039/c8nr07566b

[41] F. Vetrone , R. Naccache , A. Zamarron , A. J. de la Fuente , F. Sanz-Rodriguez , L. M. Maestro , E. M. Rodriguez , D. Jaque , J. G. Sole , J. A. Capobianco , ACS Nano 2010, 4, 3254–3258. 20441184 10.1021/nn100244a

[42] C. D. S. Brites , S. Balabhadra , L. D. Carlos , Adv. Opt. Mater. 2019, 7.

[43] Y. Li , Polyhedron 2020, 179.

[44] Y. Yang , Y. Z. Wang , Y. Feng , X. R. Song , C. Cao , G. L. Zhang , W. S. Liu , Talanta 2020, 208, 6. 10.1016/j.talanta.2019.120354 31816801

[45] D. Zhao , H. Z. Wang , G. D. Qian , CrystEngComm 2018, 20, 7395–7400.

[46] K. Miyata , Y. Konno , T. Nakanishi , A. Kobayashi , M. Kato , K. Fushimi , Y. Hasegawa , Angew. Chem. Int. Ed. 2013, 52, 6413–6416; 10.1002/anie.201301448 23649825

[47] M. O. Rodrigues , J. D. L. Dutra , L. A. O. Nunes , G. F. de Sa , W. M. de Azevedo , P. Silva , F. A. A. Paz , R. O. Freire , S. A. Junior , J. Phys. Chem. C 2012, 116, 19951–19957.

[48] C. V. Rodrigues , L. L. Luz , J. D. L. Dutra , S. A. Junior , O. L. Malta , C. C. Gatto , H. C. Streit , R. O. Freire , C. Wickleder , M. O. Rodrigues , Phys. Chem. Chem. Phys. 2014, 16, 14858–14866. 24924492 10.1039/c4cp00405a

[49] R. M. Supkowski , W. D. Horrocks , Inorg. Chim. Acta 2002, 340, 44–48.

[50] A. Beeby , I. M. Clarkson , R. S. Dickins , S. Faulkner , D. Parker , L. Royle , A. S. de Sousa , J. A. G. Williams , M. Woods , J. Chem. Soc. Perkin Trans. 2 1999, 3, 493–503.

[51] Y. Zhang , L. Chen , Z. Liu , W. Liu , M. Yuan , J. Shu , N. Wang , L. He , J. Zhang , J. Xie , X. Chen , J. Diwu , ACS Appl. Mater. Interfaces 2020, 14, 16648–16654. 10.1021/acsami.0c02783 32212614

[52] R. G. Geitenbeek , A. E. Nieuwelink , T. S. Jacobs , B. B. V. Salzmann , J. Goetze , A. Meijerink , B. M. Weckhuysen , ACS Catal. 2018, 8, 2397–2401. 29527404 10.1021/acscatal.7b04154 PMC5839602

[53] T. Hartman , R. G. Geitenbeek , C. S. Wondergem , W. van der Stam , B. M. Weckhuysen , ACS Nano 2020, 14, 3725–3735. 32307982 10.1021/acsnano.9b09834 PMC7199205

[54] C. P. Xu , R. Q. Fang , R. Luque , L. Y. Chen , Y. W. Li , Coord. Chem. Rev. 2019, 388, 268–292.

