# Supplementary material for: A Highly Stable Yttrium Organic Framework as a Host for Optical Thermometry and D2O Detection
Source: Chemistry. 2022 Mar 4;28(19):e202200410. doi: 10.1002/chem.202200410 (PMC9313560; doi:10.1002/chem.202200410)
Supplement: Supplementary file 1 — Supporting Information [file CHEM-28-0-s001.pdf]

# Chemistry–A European Journal

Supporting Information


## A Highly Stable Yttrium Organic Framework as a Host for Optical Thermometry and $D_2O$ Detection


Thomas W. Chamberlain, Rafael V. Perrella, Tamires M. Oliveira, Paulo C. de Sousa Filho,* and Richard I. Walton*

# Supporting Information


Page

1. ADDITIONAL DATA ……………………………………………………………………………….

1.1. TGA and XRD …………………………………………………………………………….. S-2

1.2. Luminescence spectra ……………………………………………………………………. S-4

1.3. Quantum yields ………………………………………………………………………… S-5

1.4. Temperature dependence of decay times for $(Y,Eu)_6$- and $(Y,Tb)_6$-MOFs ………….. S-6

1.5. Time-resolved luminescence spectra and energy transfer mechanisms …………... S-7

1.6. Thermometric performance ……………………………………………………………. S-8

1.7. Temperature dependence of decay times for mixed (Y,Eu,Tb)-MOFs ………………. S-18

1.8. Reversibility of decay times towards $D_2O$ exposure ………..……………………… S-22

2. REFERENCES ………………………………………………………………………………. S-24

# 1. ADDITIONAL DATA

## 1.1. TGA and XRD

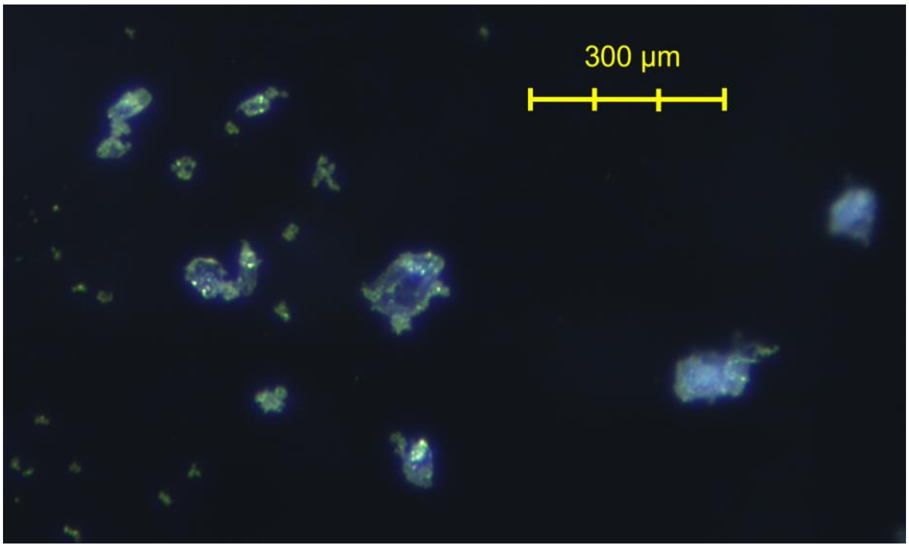


**Figure S1.** Optical microscope image of crystals of $(Y_{0.89}Tb_{0.10}Eu_{0.01})_6(BDC)_7(OH)_4(H_2O)_4$ illustrating the large, micron sized crystals formed during the synthesis. A distribution of approximately 10 µm up to around 150 µm were observed.

**Table S1.** Comparison between the calculated and measured mass losses in the TGA of $Y_6(BDC)_7(OH)_4(H_2O)_4$ and $(Y_{0.89}Tb_{0.10}Eu_{0.01})_6(BDC)_7(OH)_4(H_2O)_4$ MOFs. Calculated values were based on the empirical formulae of the MOFs, and these are shown for each stage.

| Material | | T / °C | Mass / % | Empirical formula |
|---|---|---|---|---|
| $Y_6$-MOF | Calculated | 25 | 100 | $Y_6(BDC)_7(OH)_4(H_2O)_4$ |
| | | 300 | 96 | $Y_6(BDC)_7(OH)_4$ |
| | | 1000 | 37 | $Y_2O_3$ |
| | Measured | 25 | 100 | $Y_6(BDC)_7(OH)_4(H_2O)_4$ |
| | | 300 | 94 | $Y_6(BDC)_7(OH)_4$ |
| | | 1000 | 37 | $Y_2O_3$ |
| $(Y,Eu,Tb)_6$-MOF | Calculated | 25 | 100 | $(Y_{0.89}Tb_{0.10}Eu_{0.01})_6(BDC)_7(OH)_4(H_2O)_4$ |
| | | 300 | 96 | $(Y_{0.89}Tb_{0.10}Eu_{0.01})_6(BDC)_7(OH)_4$ |
| | | 1000 | 39 | $(Y_{0.89}Tb_{0.10}Eu_{0.01})_2O_3$ |
| | Measured | 25 | 100 | $(Y_{0.89}Tb_{0.10}Eu_{0.01})_6(BDC)_7(OH)_4(H_2O)_4$ |
| | | 300 | 95 | $(Y_{0.89}Tb_{0.10}Eu_{0.01})_6(BDC)_7(OH)_4$ |
| | | 1000 | 41 | $(Y_{0.89}Tb_{0.10}Eu_{0.01})_2O_3$ |

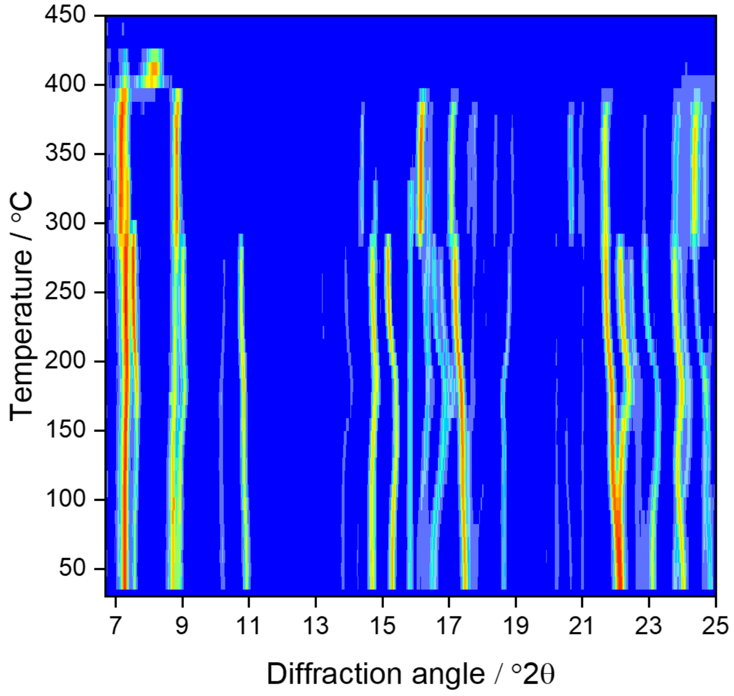


**Figure S2.** *In situ* XRD measured at 10°C intervals on heating of $(Y_{0.89}Tb_{0.10}Eu_{0.01})_6(BDC)_7(OH)_4(H_2O)_4$ from 30-450° in air. The MOF sample was held at each temperature while XRD data was recorded. * XRD peak due to appearance of sample holder as MOF decomposed.

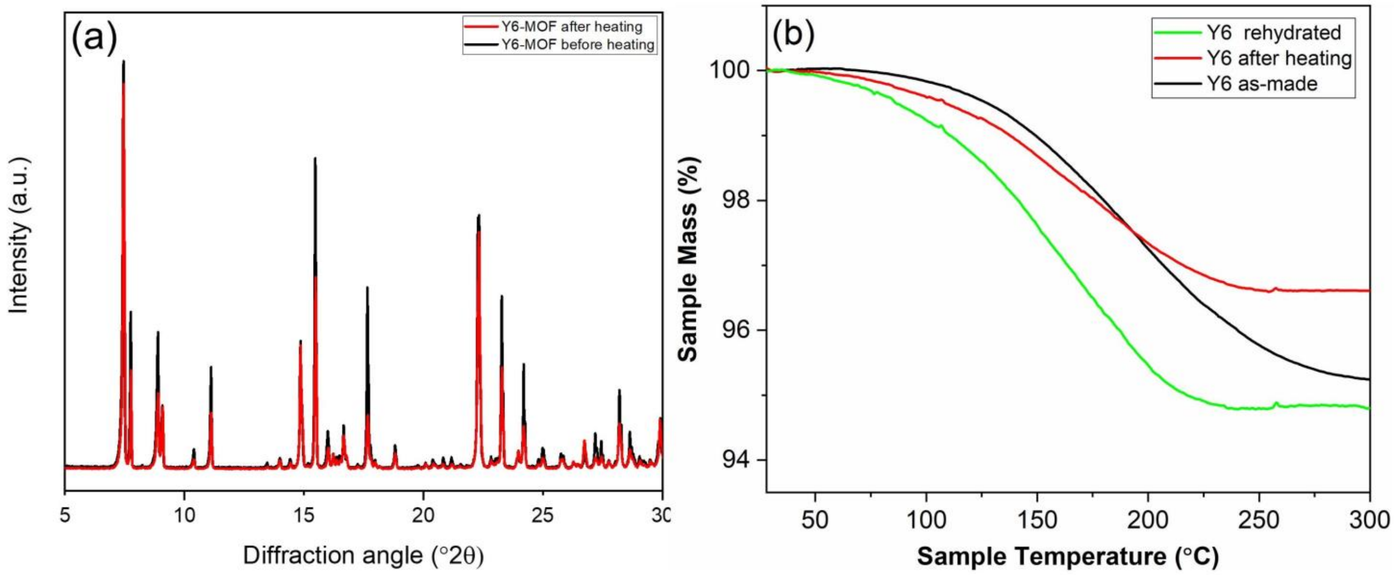


**Figure S3.** (a) Powder XRD patterns of $(Y_{0.89}Tb_{0.10}Eu_{0.01})_6(BDC)_7(OH)_4(H_2O)_4$ before and after two heating cycles to 300 °C. The material was heated directly to 300 °C where it was held for 1 hour, it was then cooled before being heated to 300 °C again and held for another 1 hour. The powder XRD data was recorded after the sample had cooled for the second time. (b) Thermogravimetric analysis of the as-made sample, after heating and after rehydration, showing how water content can be regained following dehydration.

## 1.2. Luminescence spectra

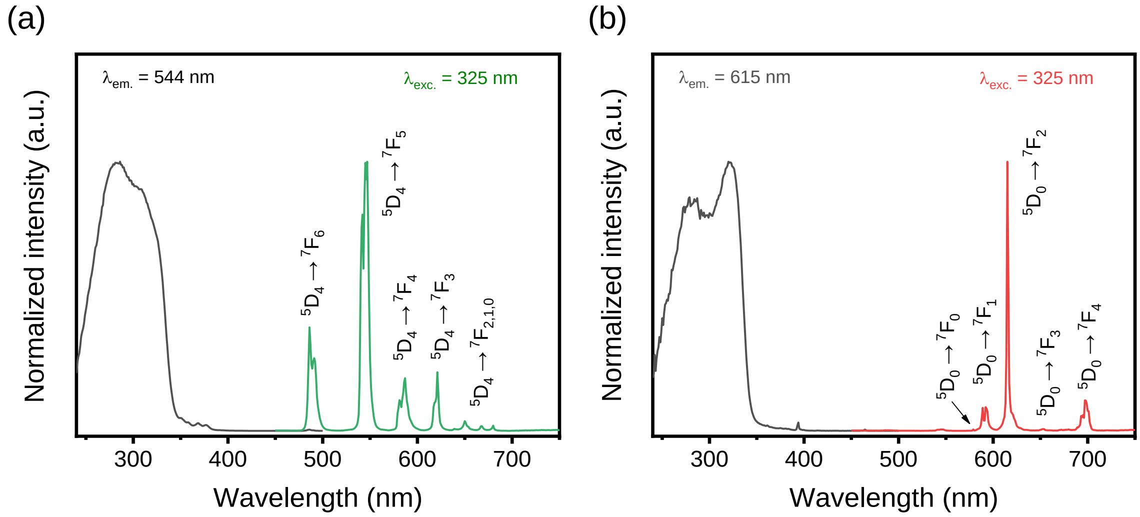


**Figure S4.** Normalized excitation (black line) and emission ($\lambda_{exc}$ = 325 nm) spectra collected at 298 K for the as-prepared (a) $(Y_{0.90}Tb_{0.10})_6(BDC)_7(OH)_4(H_2O)_4$ and (b) $(Y_{0.99}Eu_{0.01})_6(BDC)_7(OH)_4(H_2O)_4$ MOFs.

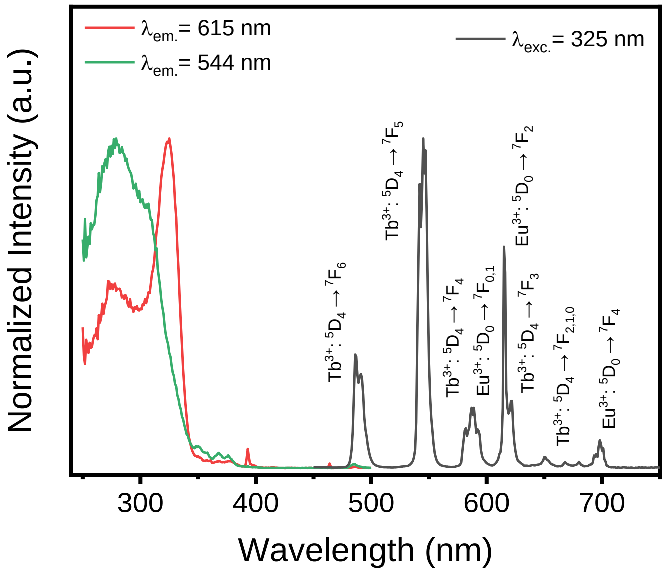


**Figure S5.** Normalized excitation and emission ($\lambda_{exc}$ = 325 nm, black line) spectra collected at 298 K for the as-prepared $(Y_{0.89}Tb_{0.10}Eu_{0.01})_6(BDC)_7(OH)_4(H_2O)_4$ MOF.

### 1.3. Quantum yields

**Table S2.** Overall quantum yield (Φ) of the as-prepared $(Y_{0.90}Tb_{0.10})_6(BDC)_7(OH)_4(H_2O)_4$, $(Y_{0.99}Eu_{0.01})_6(BDC)_7(OH)_4(H_2O)_4$ and $(Y_{0.89}Tb_{0.10}Eu_{0.01})_6(BDC)_7(OH)_4(H_2O)_4$ MOFs in powder form. Three measurements were made for each sample so that the average value is reported. The method is accurate within 10%

| | Φ (%) |
|---|---|
| $(Y_{0.90}Tb_{0.10})_6(BDC)_7(OH)_4(H_2O)_4$ | 26 |
| $(Y_{0.99}Eu_{0.01})_6(BDC)_7(OH)_4(H_2O)_4$ | 7 |
| $(Y_{0.89}Tb_{0.10}Eu_{0.01})_6(BDC)_7(OH)_4(H_2O)_4$ | 17 |

### 1.4. Temperature dependence of decay times for $(Y,Eu)_6$- and $(Y,Tb)_6$-MOFs

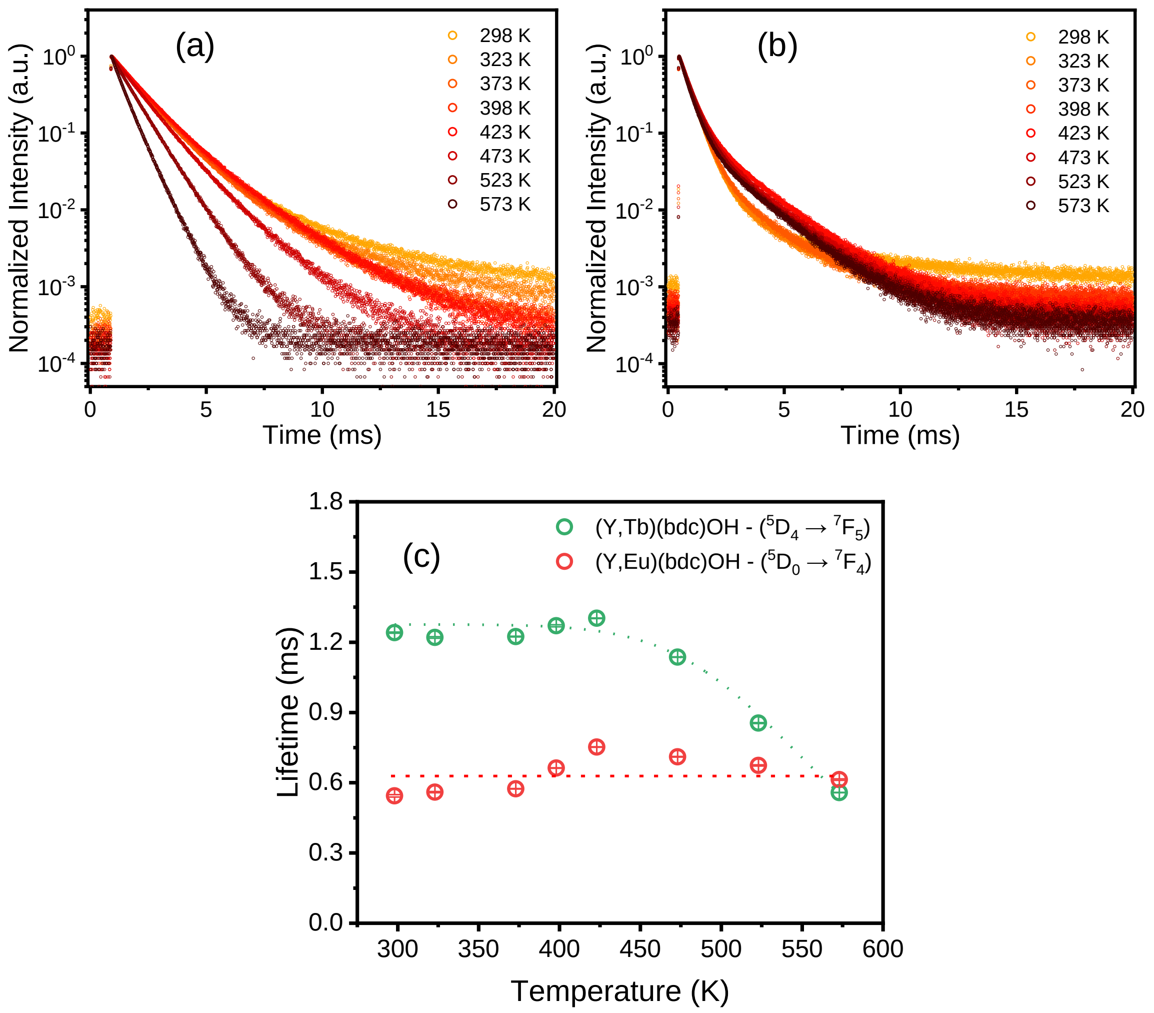


**Figure S6.** Temperature-dependent emission decay curves for (a) $(Y_{0.90}Tb_{0.10})_6(BDC)_7(OH)_4(H_2O)_4$ ($\lambda_{exc}$ = 325 nm, $\lambda_{em}$ = 544 nm, $^5D_4 \rightarrow {}^7F_5$) and (b) $(Y_{0.99}Eu_{0.01})_6(BDC)_7(OH)_4(H_2O)_4$ ($\lambda_{exc}$ = 325 nm, $\lambda_{em}$ = 698 nm, $^5D_0 \rightarrow {}^7F_4$) MOFs. (c) Dependence of the $^5D_4$ ($Tb^{3+}$) and $^5D_0$ ($Eu^{3+}$) lifetimes against temperature from 298 to 573 K.

### 1.5. Time-resolved luminescence spectra and energy transfer mechanisms

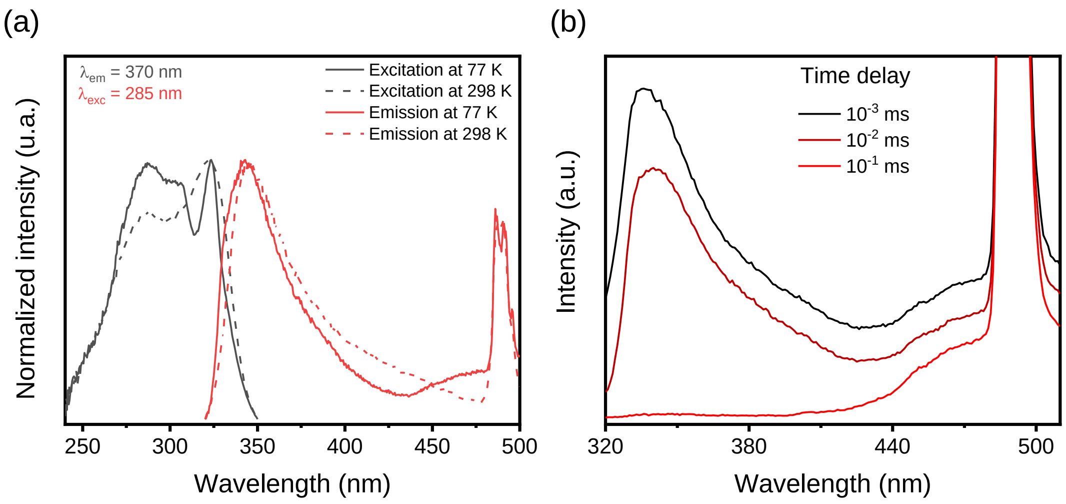


**Figure S7.** (a) Excitation (black, $\lambda_{exc}$ = 370 nm) and emission (red, $\lambda_{exc}$ = 285 nm) spectra of the lowed-doped $(Y_{0.90}Tb_{0.10})_6(BDC)_7(OH)_4(H_2O)_4$ MOF showing the BDC ligand signals. The spectra were recorded at 298 K (dotted line) and 77 K (solid line). (b) Time-resolved emission spectra obtained at 77 K in the $10^{-3}$ – $10^{-1}$ ms range.

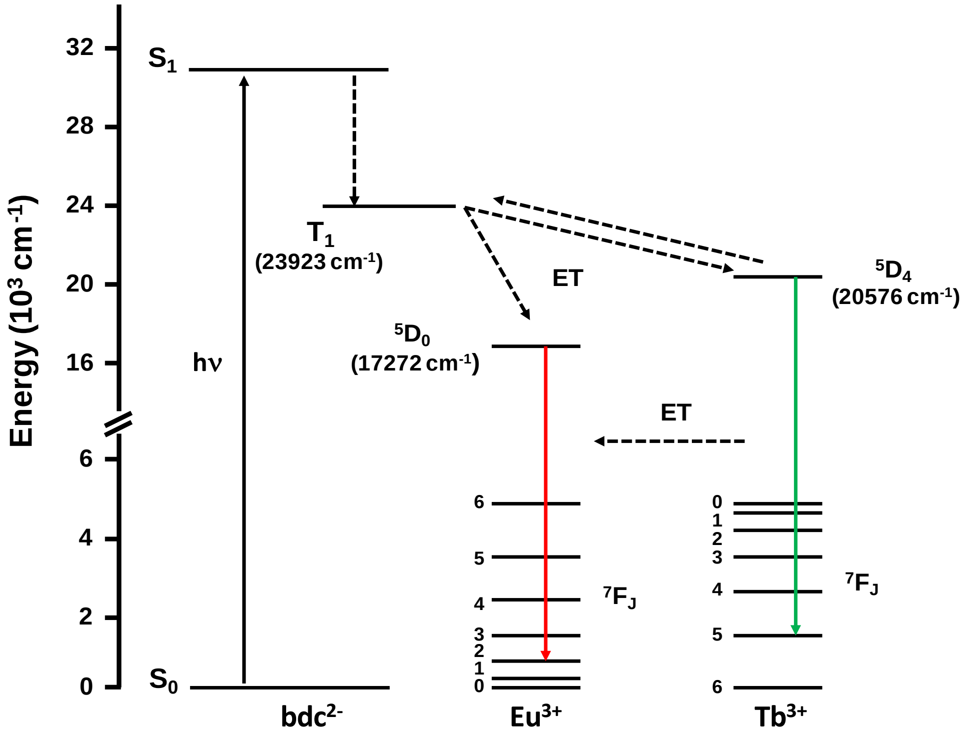


**Figure S8.** Schematic energy levels diagram representing the energy transfer mechanisms involved in the luminescence of $(Y_{0.89}Tb_{0.10}Eu_{0.01})_6(BDC)_7(OH)_4(H_2O)_4$ MOF. Abbreviations: S, singlet; T, triplet; hν, energy absorption; ET, energy transfer. Solid and dotted lines correspond to radiative and non-radiative process, respectively.

### 1.6. Thermometric performance

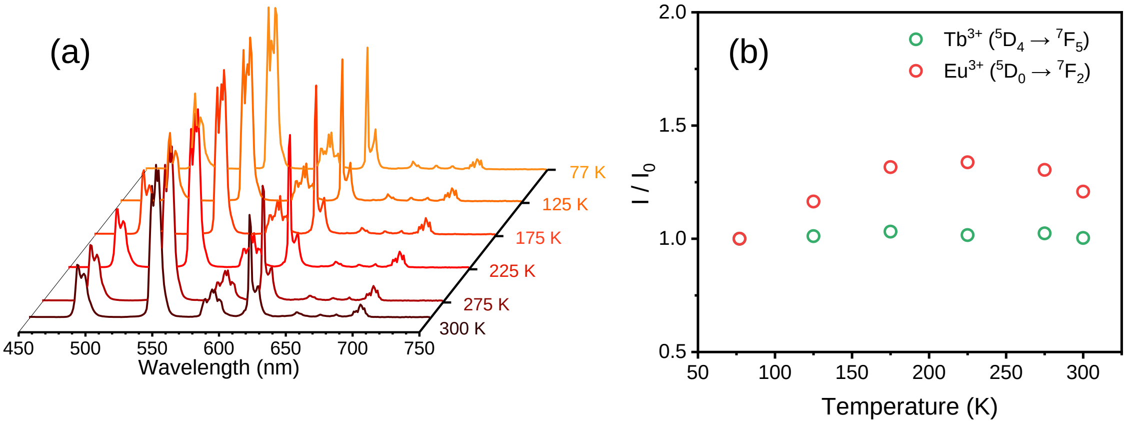


**Figure S9.** (a) Temperature-dependent emission spectra ($\lambda_{exc}$ = 325 nm, 77 – 300 K) of the as-prepared $(Y_{0.89}Tb_{0.10}Eu_{0.01})_6(BDC)_7(OH)_4(H_2O)_4$ MOF. (b) Integrated intensities of the $^5D_4 \rightarrow {}^7F_5$ (544 nm, green) and $^5D_0 \rightarrow {}^7F_2$ (610 nm, red) transitions normalized to the corresponding values obtained at 77 K ($I_0$).

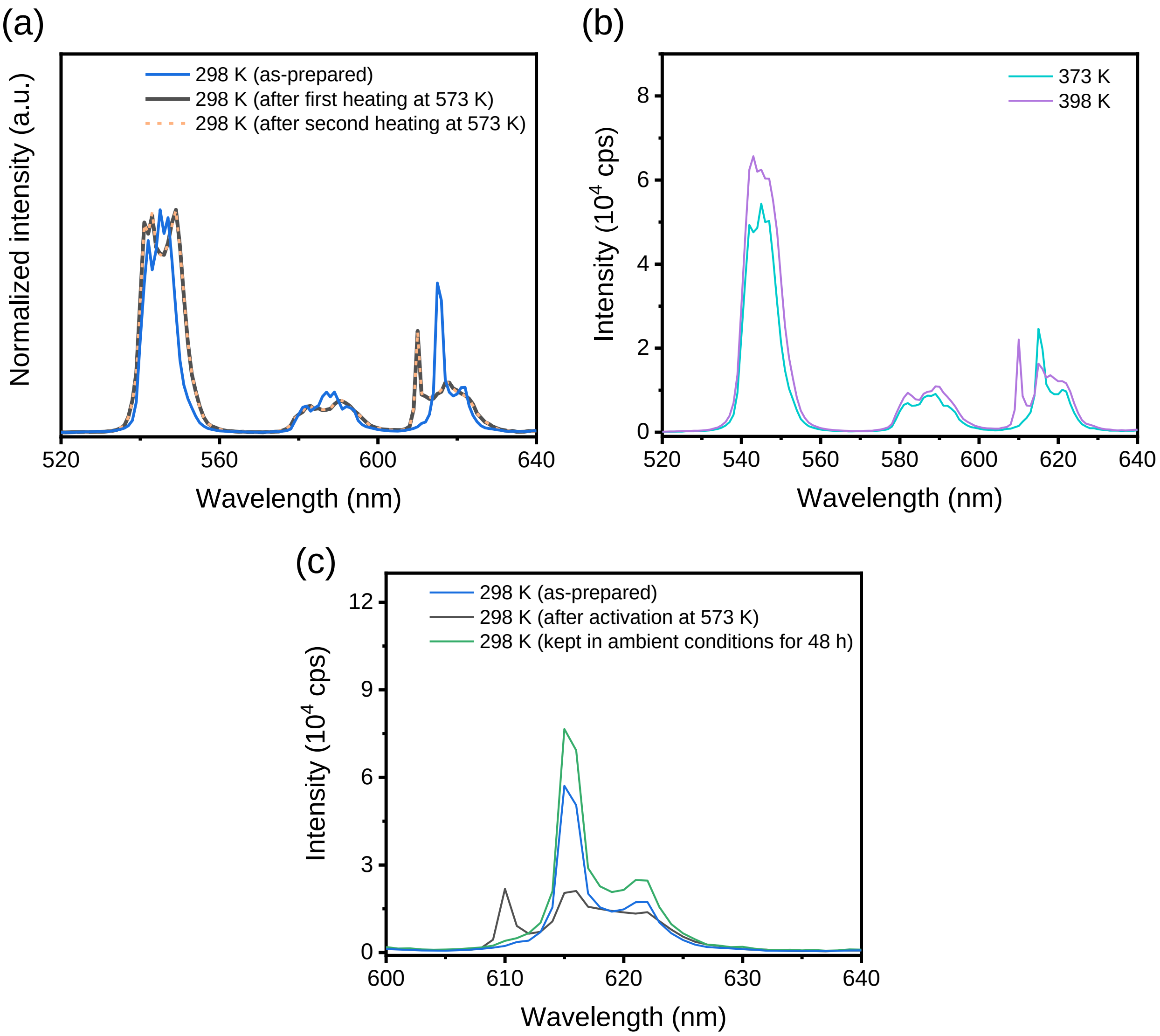


**Figure S10.** a) Emission spectra ($\lambda_{exc}$ = 325 nm) of the $(Y_{0.89}Tb_{0.10}Eu_{0.01})_6(BDC)_7(OH)_4(H_2O)_4$ MOF acquired at 298 K. The blue line refers to the as-prepared (hydrated) solid. The black and dashed orange lines refer to the activated solid after a first heating process at 573 K for 3 h (black solid line) and after a second heating at 573 K for 10 min (dashed orange line). (b) Emission spectra collected at 373 K (cyan) and 398 K (violet) showing the arising of the signal at 610 nm. (c) Emission spectra (600 – 640 nm range) of the LnMOF as-prepared (blue line), activated (black), and kept in ambient conditions for 48 h (green), indicating the reversibility of the hydration-dehydration process.

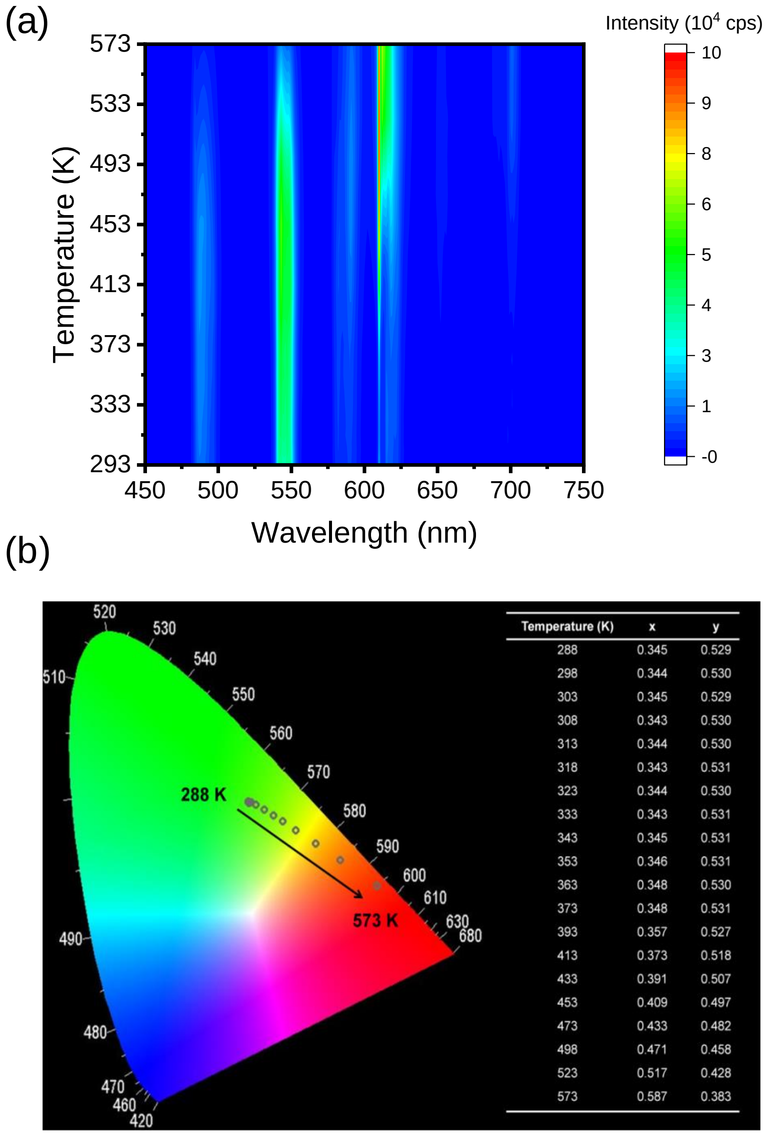


| Temperature (K) | x | y |
|---|---|---|
| 288 | 0.345 | 0.529 |
| 298 | 0.344 | 0.530 |
| 303 | 0.345 | 0.529 |
| 308 | 0.343 | 0.530 |
| 313 | 0.344 | 0.530 |
| 318 | 0.343 | 0.531 |
| 323 | 0.344 | 0.530 |
| 333 | 0.343 | 0.531 |
| 343 | 0.345 | 0.531 |
| 353 | 0.346 | 0.531 |
| 363 | 0.348 | 0.530 |
| 373 | 0.348 | 0.531 |
| 393 | 0.357 | 0.527 |
| 413 | 0.373 | 0.518 |
| 433 | 0.391 | 0.507 |
| 453 | 0.409 | 0.497 |
| 473 | 0.433 | 0.482 |
| 498 | 0.471 | 0.458 |
| 523 | 0.517 | 0.428 |
| 573 | 0.587 | 0.383 |

**Figure S11.** (a) Dependence of the emission intensities ($\lambda_{exc}$ = 325 nm) of the powder $(Y_{0.89}Tb_{0.10}Eu_{0.01})_6(BDC)_7(OH)_4(H_2O)_4$ MOF against temperature in the 288 – 573 K range. (b) Chromaticity diagram representing the evolution of the emission colour in the same temperature range. Inset table shows CIE 1931 (x,y) chromaticity coordinates.

Based on the temperature-dependent emission spectra illustrated in Figure S11a, the Δ parameter can be linearly related to the temperature between 283 and 343 K according to the Equation (S1):

$$\Delta(T) = 0.0077\,T - 0.1726 \qquad \text{(S1)}$$

where, Δ is defined as the integrated intensity ratio between the $^5D_4 \rightarrow {}^7F_5$ ($Tb^{3+}$, 544 nm) and $^5D_0 \rightarrow {}^7F_2$ ($Eu^{3+}$, 615 nm) transitions and T is the absolute temperature.

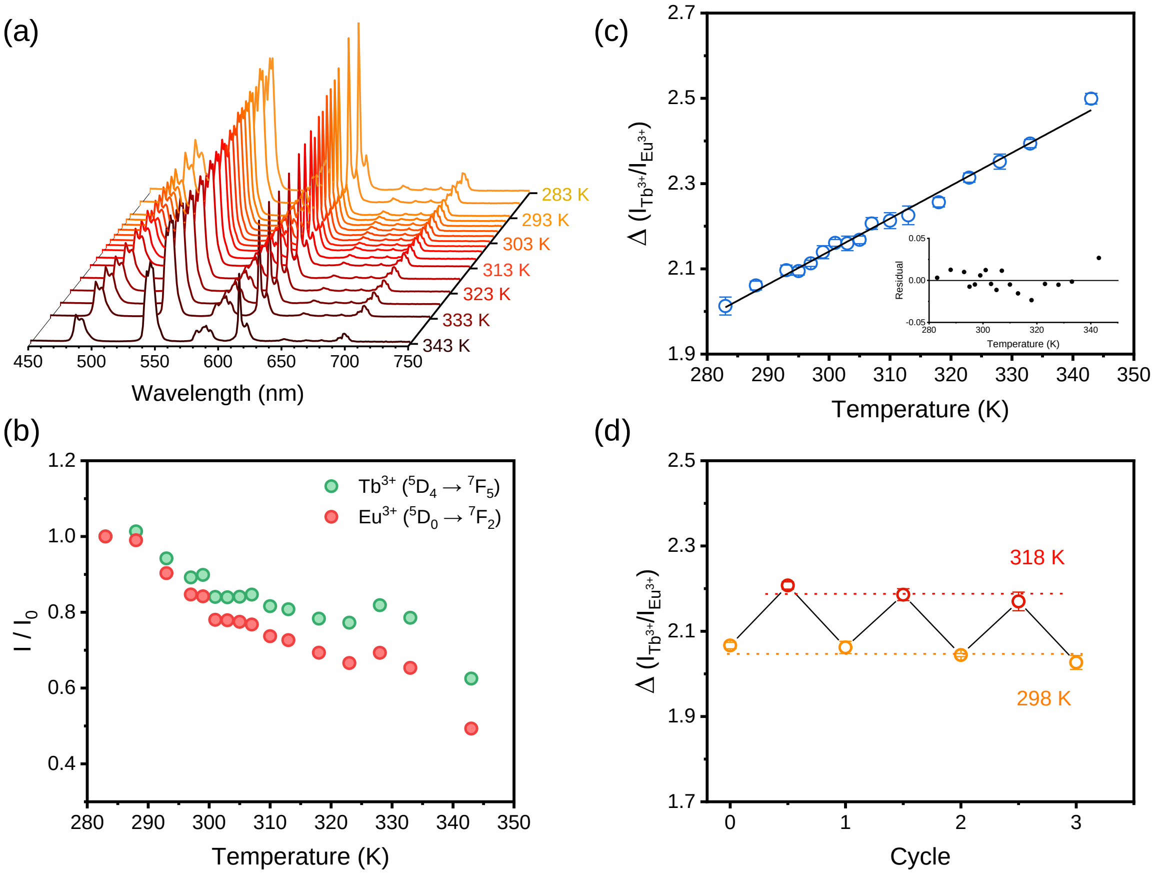


**Figure S12.** (a) Temperature-dependent emission spectra ($\lambda_{exc}$ = 325 nm, 283 – 343 K) of the activated $(Y_{0.89}Tb_{0.10}Eu_{0.01})_6(BDC)_7(OH)_4(H_2O)_4$ MOF as water suspension (1 g $L^{-1}$); (b) integrated intensities of the $^5D_4 \rightarrow {}^7F_5$ (544 nm, green) and $^5D_0 \rightarrow {}^7F_2$ (615 nm, red) transitions normalized to the corresponding values obtained at 283 K ($I_0$); (c) dependence of the Δ parameter on the temperature. The solid line is the best fit to the experimental data ($r^2$ > 0.990) using Equation (S1), the inset shows the residuals of the fit; (d) repeatability test for the thermometric parameter performed under temperature cycling between 298 and 318 K.

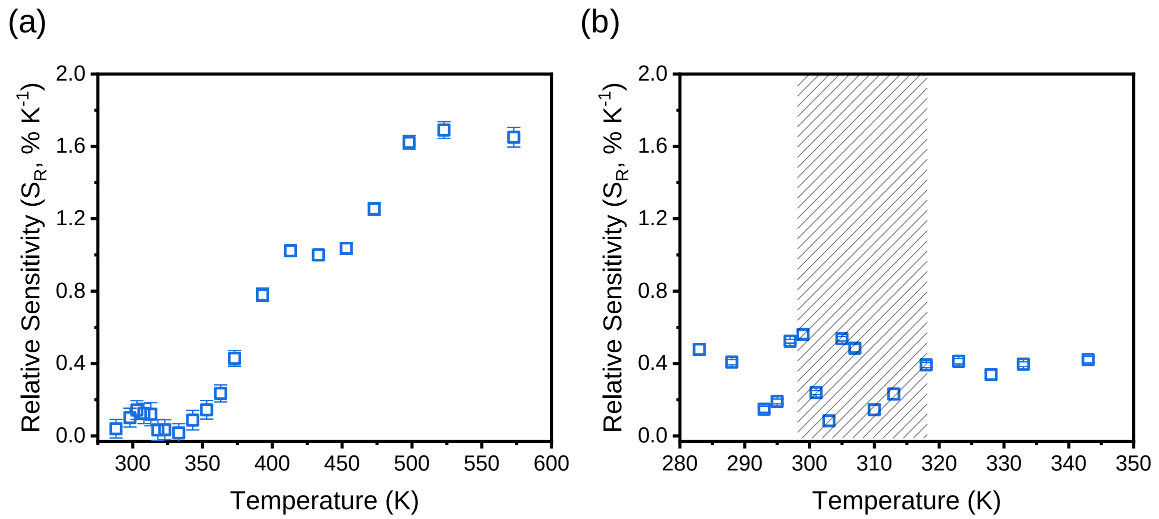


**Figure S13.** Relative thermal sensitivity ($S_R$) for the activated $(Y_{0.89}Tb_{0.10}Eu_{0.01})_6(BDC)_7(OH)_4(H_2O)_4$ MOF thermometer in (a) powder form (288 – 573 K) and (b) aqueous suspension (283 – 343 K). The physiological temperature range (298-318 K) is shadowed in (b).

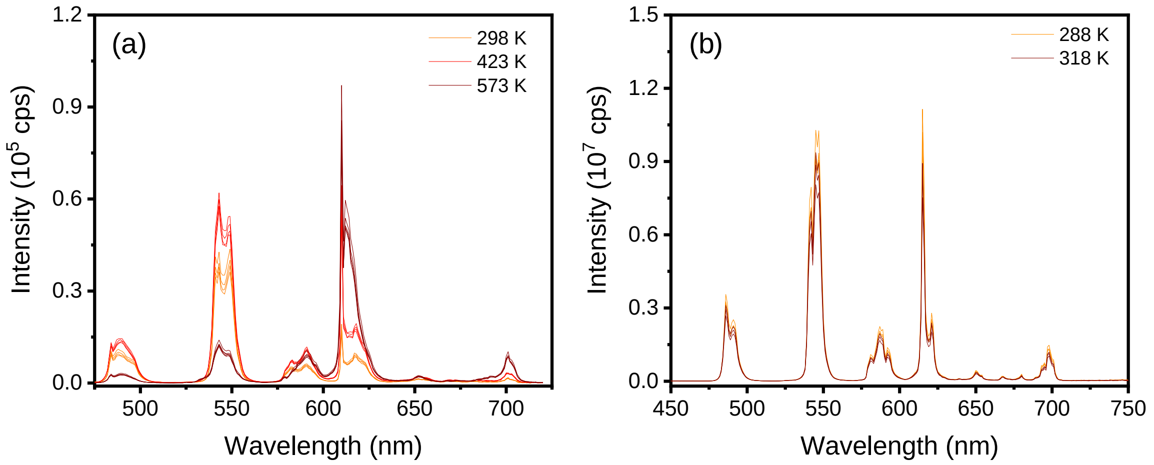


**Figure S14.** Emission spectra of the activated $(Y_{0.89}Tb_{0.10}Eu_{0.01})_6(BDC)_7(OH)_4(H_2O)_4$ MOF ($\lambda_{exc}$ = 325 nm) at different temperatures demonstrating the repeatability of the thermometric parameter, Δ, upon heating-cooling cycles for (a) solids (five cycles, 298 – 573 K) and (b) water suspension (three cycles, 288 – 318 K).

**Table S3.** Comparing the performance of some Ln-based MOFs luminescent ratiometric thermometers in terms of ligand, composition, temperature range, maximum relative sensitivity ($S_m$) and corresponding temperature ($T_m$), and optical parameter

| Ligand (acid form) | Composition | Range (K) | $S_m$ (% $K^{-1}$) | $T_m$ (K) | Optical parameter | Ref |
|---|---|---|---|---|---|---|
| 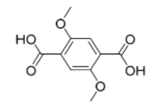 | $Tb_{0.9931}Eu_{0.0069}$(dmbdc) | 50-200 | 1.15 | 200 | $I_{Tb}/I_{Eu}$ [b] | 2 |
| | $Tb_{0.9954}Eu_{0.0046}$(dmbdc) | | 0.61 | 200 | | |
| | $Tb_{0.9989}Eu_{0.0011}$(dmbdc) | | 0.52 | 200 | | |
| 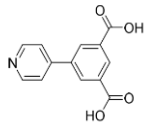 | $Tb_{0.99}Eu_{0.01}$(pia) | 100-300 | 2.75 | 300 | $I_{Tb}/I_{Eu}$ [b] | 3 |
| | $Tb_{0.90}Eu_{0.10}$(pia) | 100-300 | 3.27 | 300 | | |
| | $Tb_{0.95}Eu_{0.05}$(pia) | 100-300 | 2.48 | 250 | | |
| | $Tb_{0.50}Eu_{0.50}$(pia) | 75-275 | 2.02 | 275 | | |
| 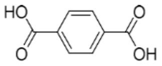 | $(Tb_{0.99}Eu_{0.01})_2(bdc)_3(H_2O)_4$ | 290-320 | 0.31 [a] | 318 | $I_{Tb}/I_{Eu}$ [b] | 4 |
| 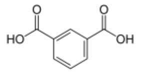 | $[(Tb_{0.90}Eu_{0.10})_2$(1,3-bdc)$_3(H_2O)_2].H_2O$ | 12-101 | 3.30 | 36 | $I_{Tb}/I_{Eu}$ [b] | 5 |
| 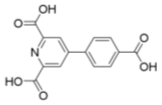 | $Tb_{0.957}Eu_{0.043}$(cpda) | 40-300 | 16 | 300 | $I_{Tb}/I_{Eu}$ [b] | 6 |
| 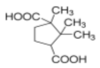 D-$H_2$cam 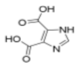 $H_3$imdc | $(Tb_{0.3}Eu_{0.7})_2$(D-cam)(Himdc)$_2(H_2O)_2$ | 100-450 | 0.11 | 450 | $I_{Tb}/I_{Eu}$ [b] | 7 |
| 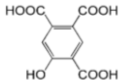 | $Tb_{0.95}Eu_{0.05}$HL | 4-300 | 31 | 4 | $I_{Tb}/I_{Eu}$ [b] | 8 |
| 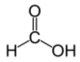 | $Sr(HCOO)_2:Eu^{2+}/Eu^{3+}$ | 9-293 | 3.8 | 293 | $I_{Eu}^{2+}/I_{Eu}^{3+}$ [c] | 9 |
| 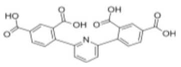 | $[(CH_3)_2NH_2)[Tb_{0.9911}Eu_{0.0089}L(H_2O)_2]$ | 77-450 | 2.71 | 450 | $I_{Tb}/I_{Eu}$ [b] | 10 |
| | $[(CH_3)_2NH_2)[Tb_{0.9934}Eu_{0.0066}L(H_2O)_2]$ | | 3.76 | 450 | | |
| | $[(CH_3)_2NH_2)[Gd_{0.927}Tb_{0.060}Eu_{0.013}L(H_2O)_2]$ | | 6.11 | 430 | | |
| | $[(CH_3)_2NH_2][Gd_{0.882}Tb_{0.085}Eu_{0.033}L(H_2O)_2]$ | | 3.62 | 400 | | |
| 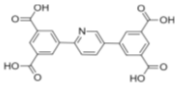 | $[(Tb_{0.81}Eu_{0.19})_2$(pddi)$(NO_3)_2(H_2O)_4]$ | 313-473 | 0.37 | 473 | $I_{Tb}/I_{Eu}$ [b] | 11 |
| 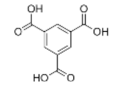 | $Y_{0.94}Dy_{0.05}Eu_{0.01}$(btc) | 80-200 | 0.64 | 170 | $I_{Dy}/I_{Eu}$ [d] | 12 |
| 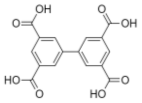 | $[(CH_3)_2NH_2]Tb_{0.964}Eu_{0.036}$(bptc) | 77-377 | 9.42 | 310 | $I_{Tb}/I_{Eu}$ [b] | 13 |
| 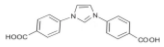 | $Tb_{0.90}Eu_{0.10}L$ | 303-423 | 1.75 | 423 | $I_{Tb}/I_{Eu}$ [b] | 14 |
| 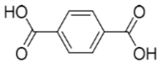 | $(Y_{0.89}Tb_{0.10}Eu_{0.01})_6(BDC)_7(OH)_4(H_2O)_4$ | 288-573 | 1.69 | 523 | $I_{Tb}/I_{Eu}$ [b] | This work |
| | | 283-343 | 0.35 [a] | const. | $I_{Tb}/I_{Eu}$ [b] | |

[a] Water suspension
[b] $I_{Tb}/I_{Eu}$ corresponds to the ratio between the $^5D_4 \rightarrow ^7F_5$ ($Tb^{3+}$) and $^5D_0 \rightarrow ^7F_2$ ($Eu^{3+}$) transitions.
[c] $I_{Eu}^{2+}/I_{Eu}^{3+}$ corresponds to the ratio between the $4f^65d^1 \rightarrow 4f^7$ ($Eu^{2+}$) and $^5D_0 \rightarrow ^7F_2$ ($Eu^{3+}$) transitions.
[d] $I_{Dy}/I_{Eu}$ corresponds to the ratio between the $^4F_{9/2} \rightarrow ^6H_{15/2}$ ($Dy^{3+}$) and $^5D_0 \rightarrow ^7F_2$ ($Eu^{3+}$) transitions.

The temperature uncertainty is given by Equation (S2):[15]

$$\delta T = \frac{1}{S_R}\frac{\delta\Delta}{\Delta} \tag{S2}$$

where $S_R$ is the relative thermal sensitivity and $\delta\Delta/\Delta$ is the relative error in the determination of the thermometric parameter. For measurements involving the water suspension, the relative errors ($\delta\Delta/\Delta$) were obtained from the standard deviation of three acquisitions. When particles in powder form are considered, the errors of the $\Delta$ parameter were calculated from fractional uncertainties ($\delta_F$), defined as the reciprocal of the signal-to-noise ratio ($R_{S/N,}$ Equation (S3)):

$$\delta_F = 1/\mathrm{R}_{S/N} \tag{S3}$$

The errors on the integrated intensities ($\delta I_{Tb}$, $\delta I_{Eu}$) were taken as the product between fractional uncertainties ($\delta_F$) and integrated intensities ($I_{Tb}$, $I_{Eu}$). For each intensity ratio, the relative error ($\delta\Delta/\Delta$) is calculated as (Equation (S4)):[15]

$$\frac{\delta\Delta}{\Delta} = \sqrt{\left(\frac{\delta \mathrm{I_{Tb}}}{\mathrm{I_{Tb}}}\right)^2 + \left(\frac{\delta \mathrm{I_{Eu}}}{\mathrm{I_{Eu}}}\right)^2} \tag{S4}$$

The labels $I_{Tb}$ and $I_{Eu}$ correspond to the integrated intensities of the $^5D_4 \rightarrow {}^7F_5$ (544 nm) and $^5D_0 \rightarrow {}^7F_0$ (610 nm) transitions.

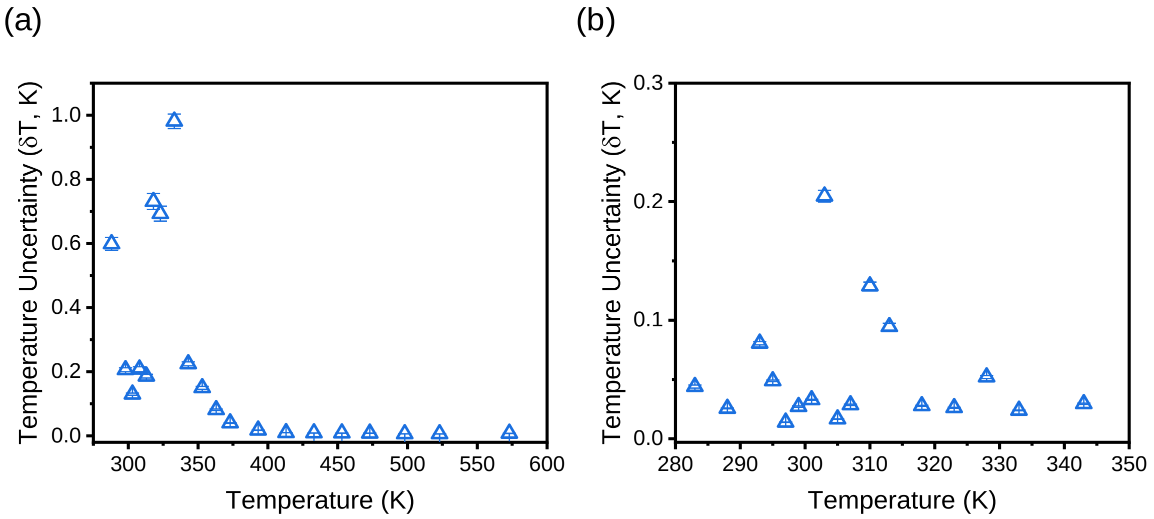


**Figure S15.** Dependence of temperature uncertainty (δT) against the temperature for activated $(Y_{0.89}Tb_{0.10}Eu_{0.01})_6(BDC)_7(OH)_4(H_2O)_4$ MOF thermometer in (a) powder form (288 – 573 K) and (b) aqueous suspension (283 – 343 K).

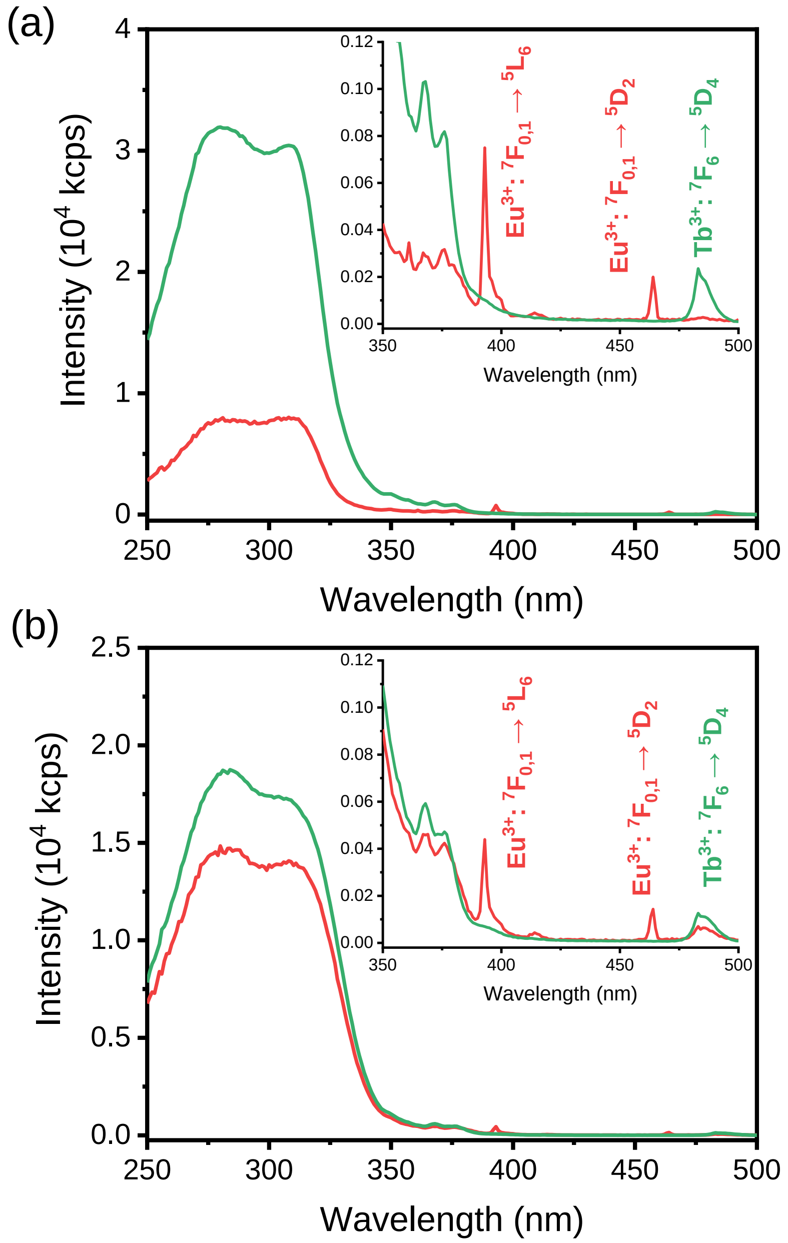


**Figure S16.** Excitation spectra recorded at (a) 298 K and (b) 473 K of the $(Y_{0.89}Tb_{0.10}Eu_{0.01})_6(BDC)_7(OH)_4(H_2O)_4$ MOF monitoring the emissions at 544 nm ($Tb^{3+}$, $^5D_4 \rightarrow {}^7F_5$, green line) and at 698 nm ($Eu^{3+}$, $^5D_0 \rightarrow {}^7F_4$).

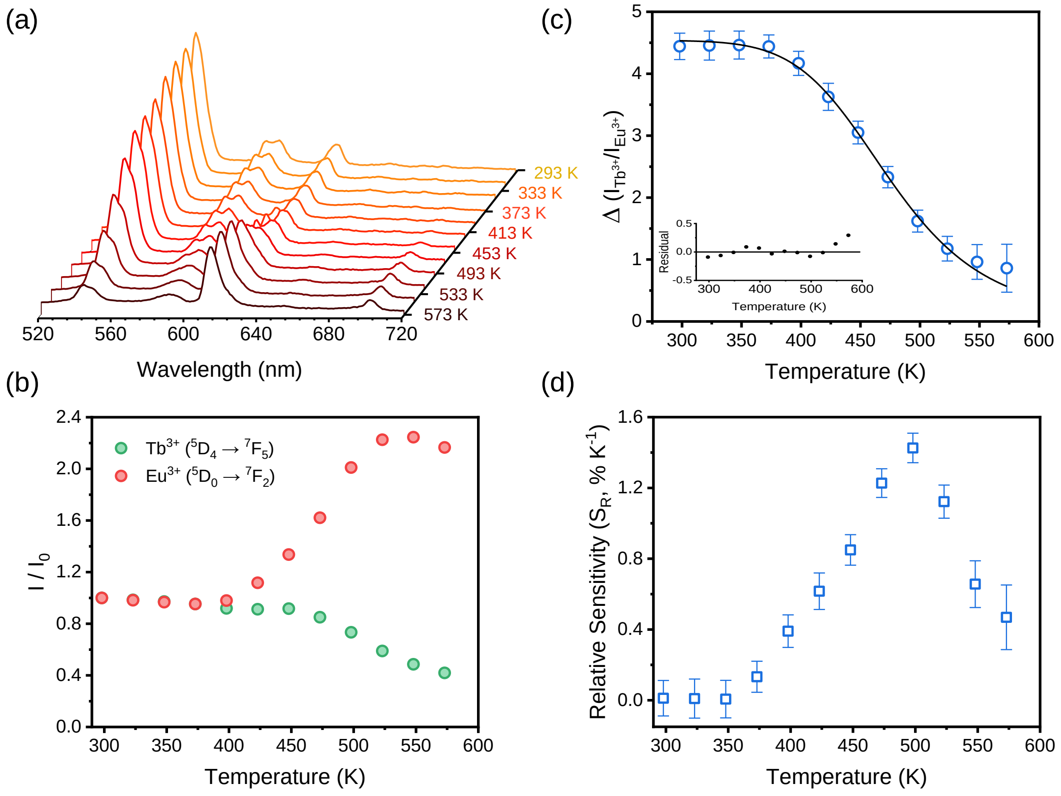


**Figure S17.** (a) Temperature-dependent emission spectra ($\lambda_{exc}$ = 486 nm, 298 – 573 K) of the activated $(Y_{0.89}Tb_{0.10}Eu_{0.01})_6(BDC)_7(OH)_4(H_2O)_4$ MOF. (b) Integrated intensities of the $^5D_4 \rightarrow {}^7F_5$ (544 nm, green) and $^5D_0 \rightarrow {}^7F_2$ (610 nm, red) transitions normalized to the corresponding values obtained at 298 K ($I_0$). (c) Dependence of the Δ parameter on the temperature. The solid line is the best fit to the experimental data ($r^2$ > 0.996) using Equation (1), the inset shows the residuals of the fit. (d) Relative thermal sensitivity, $S_R$, considering the Δ parameter.

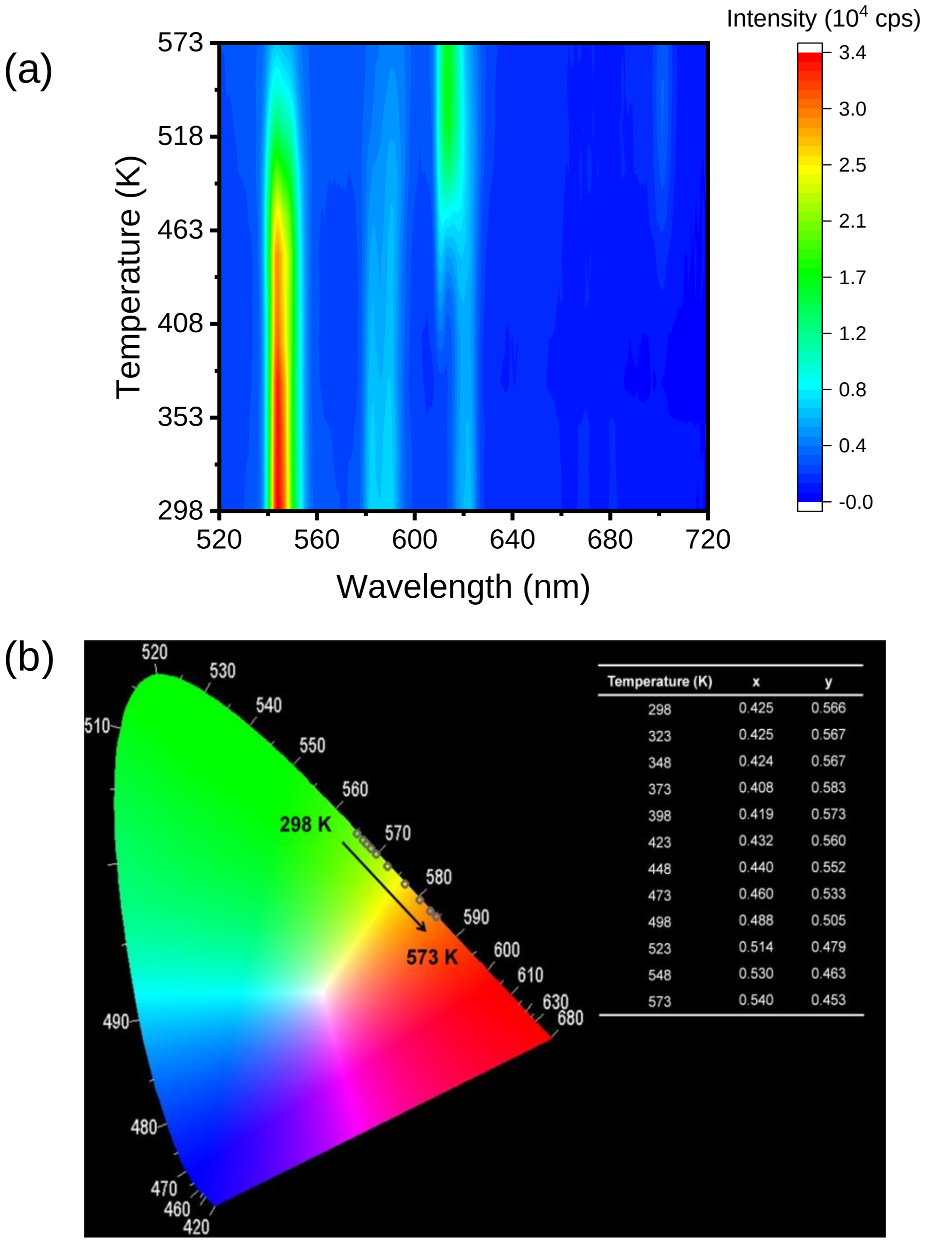


**Figure S18.** (a) Dependence of the emission intensities ($\lambda_{exc}$ = 486 nm) of the powder $(Y_{0.89}Tb_{0.10}Eu_{0.01})_6(BDC)_7(OH)_4(H_2O)_4$ MOF against temperature in the 298 – 573 K range. (b) Chromaticity diagram representing the evolution of the emission colour in the same temperature range. Inset table shows CIE 1931 (x,y) chromaticity coordinates.

## 1.7. Temperature dependence of decay times for mixed (Y,Eu,Tb)-MOFs

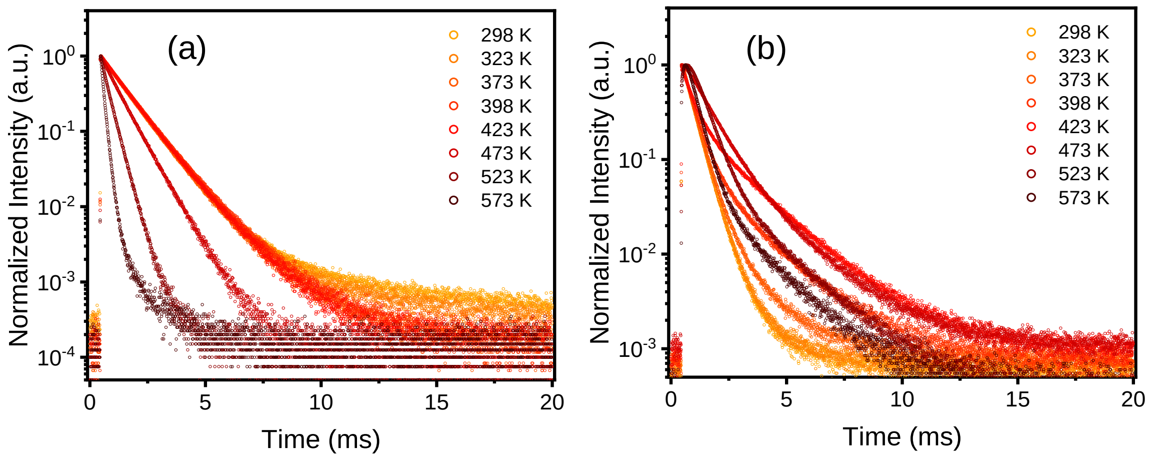


**Figure S19.** Temperature-dependent emission decay curves for the $(Y_{0.89}Tb_{0.10}Eu_{0.01})_6(BDC)_7(OH)_4(H_2O)_4$ MOF monitoring the (a) $^5D_4 \rightarrow {}^7F_5$ $Tb^{3+}$ transition ($\lambda_{exc}$ = 325 nm, $\lambda_{em}$ = 544 nm) and (b) $^5D_0 \rightarrow {}^7F_4$ $Eu^{3+}$ transition ($\lambda_{exc}$ = 325 nm, $\lambda_{em}$ = 698 nm).

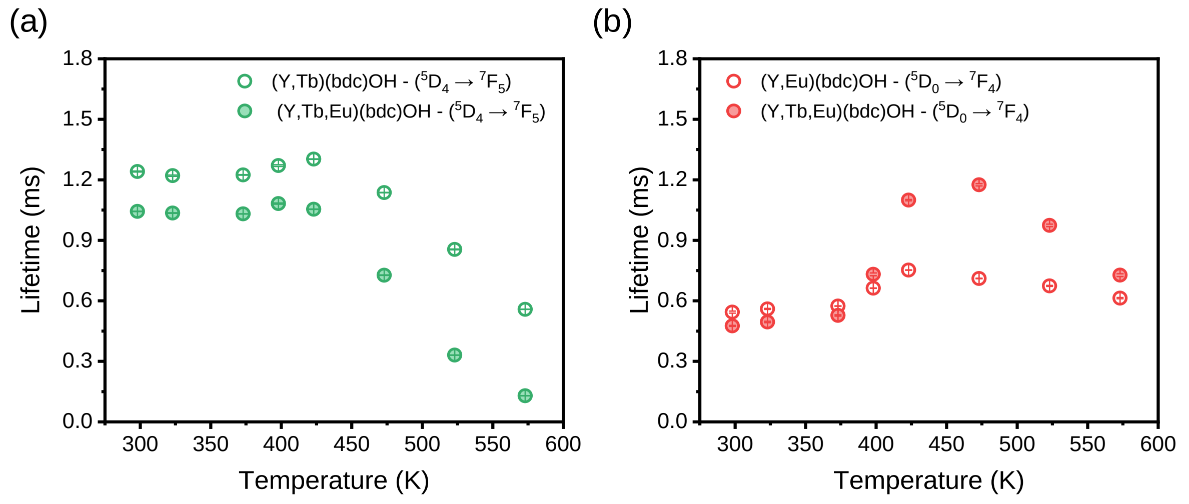


**Figure S20.** Dependence of the (a) $^5D_4$ ($Tb^{3+}$, green circles) and (b) $^5D_0$ ($Eu^{3+}$, red circles) lifetimes against temperature from 298 to 573 K for the $(Y_{0.90}Tb_{0.10})_6(BDC)_7(OH)_4(H_2O)_4$ (open green circles), $(Y_{0.99}Eu_{0.01})_6(BDC)_7(OH)_4(H_2O)_4$ (open red circles), and $(Y_{0.89}Tb_{0.10}Eu_{0.01})_6(BDC)_7(OH)_4(H_2O)_4$ (filled green/red circles) MOFs.

**Table S4.** Emission lifetimes (ms) for the $(Y_{0.90}Tb_{0.10})_6(BDC)_7(OH)_4(H_2O)_4$, $(Y_{0.99}Eu_{0.01})_6(BDC)_7(OH)_4(H_2O)_4$ and $(Y_{0.89}Tb_{0.10}Eu_{0.01})_6(BDC)_7(OH)_4(H_2O)_4$ MOFs. The $^5D_4$ ($Tb^{3+}$) and $^5D_0$ ($Eu^{3+}$) luminescence lifetimes were obtained monitoring the $^5D_4 \rightarrow {}^7F_5$ (544 nm, $Tb^{3+}$) and $^5D_0 \rightarrow {}^7F_4$ (698 nm, $Eu^{3+}$) transitions, respectively

| Temperature | $(Y_{0.90}Tb_{0.10})_6(BDC)_7(OH)_4(H_2O)_4$ $^5D_4$ ($Tb^{3+}$) | $(Y_{0.99}Eu_{0.01})_6(BDC)_7(OH)_4(H_2O)_4$ $^5D_0$ ($Eu^{3+}$) | $(Y_{0.89}Tb_{0.10}Eu_{0.01})_6(BDC)_7(OH)_4(H_2O)_4$ $^5D_4$ ($Tb^{3+}$) | $(Y_{0.89}Tb_{0.10}Eu_{0.01})_6(BDC)_7(OH)_4(H_2O)_4$ $^5D_0$ ($Eu^{3+}$) |
|---|---|---|---|---|
| 298 K | 1.241 ± 0.002 | 0.544 ± 0.005 | 1.043 ± 0.002 | 0.476 ± 0.003 |
| 323 K | 1.221 ± 0.004 | 0.560 ± 0.003 | 1.035 ± 0.002 | 0.495 ± 0.004 |
| 373 K | 1.224 ± 0.003 | 0.574 ± 0.001 | 1.031 ± 0.001 | 0.528 ± 0.004 |
| 398 K | 1.270 ± 0.005 | 0.663 ± 0.002 | 1.082 ± 0.003 | 0.731 ± 0.007 |
| 423 K | 1.302 ± 0.002 | 0.752 ± 0.001 | 1.054 ± 0.002 | 1.099 ± 0.004 |
| 473 K | 1.137 ± 0.002 | 0.711 ± 0.002 | 0.727 ± 0.002 | 1.175 ± 0.006 |
| 523 K | 0.855 ± 0.002 | 0.674 ± 0.003 | 0.331 ± 0.001 | 0.974 ± 0.006 |
| 573 K | 0.558 ± 0.001 | 0.613 ± 0.004 | 0.130 ± 0.001 | 0.727 ± 0.007 |

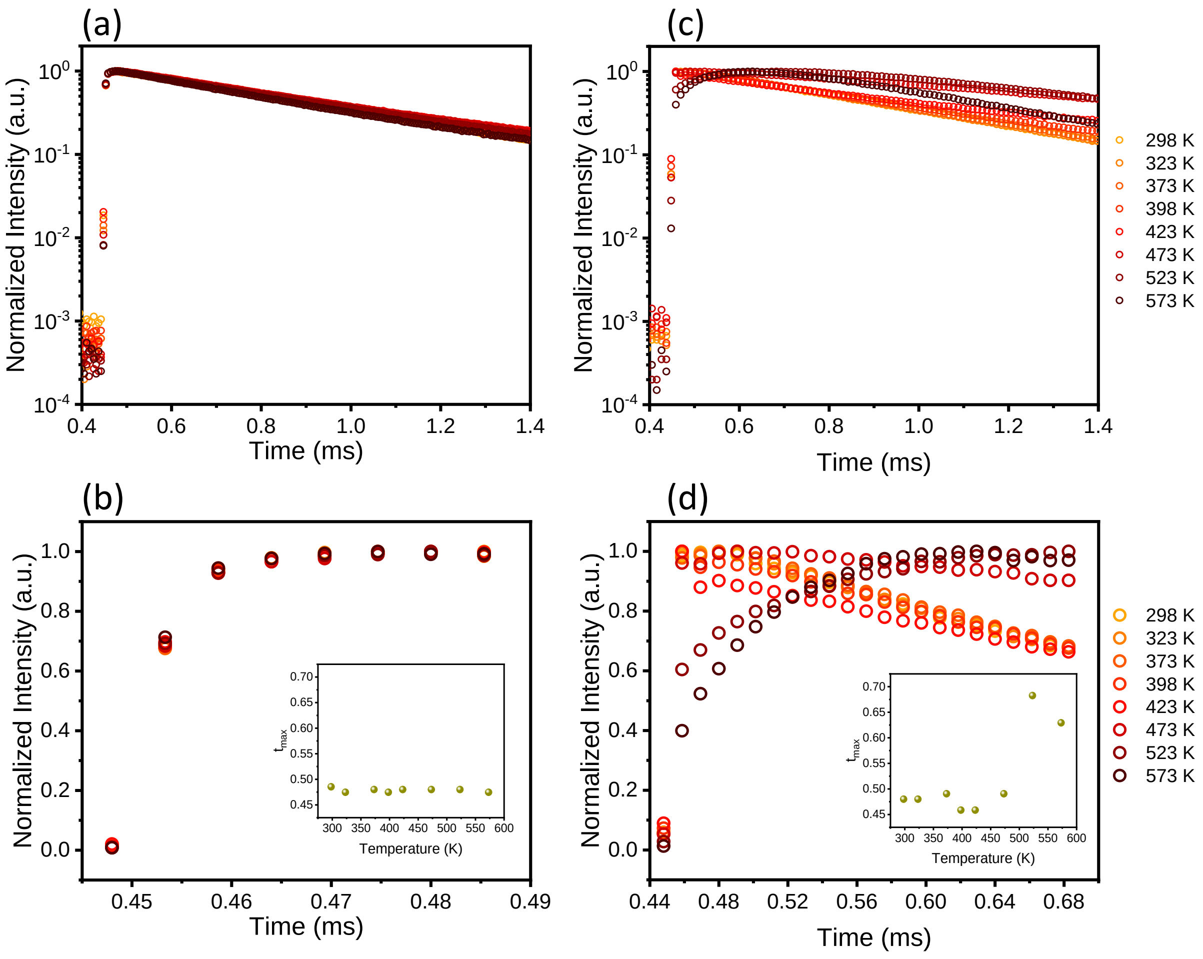


**Figure S21.** Emission intensities vs. times at different temperatures monitoring the $^5D_0 \rightarrow {}^7F_4$ $Eu^{3+}$ transition ($\lambda_{exc}$ = 325 nm, $\lambda_{em}$ = 698 nm) for the (a,b) $(Y_{0.99}Eu_{0.01})_6(BDC)_7(OH)_4(H_2O)_4$ and (c,d) $(Y_{0.89}Tb_{0.10}Eu_{0.01})_6(BDC)_7(OH)_4(H_2O)_4$ MOFs. (b) and (d) show amplifications of the regions comprising the rise times of the $^5D_0$ state. The insets in (b) and (d) correspond to the time elapsed before emission intensities reach the global maxima ($t_{max}$) before the decays start for the different temperatures.

**Table S5.** $^5D_0$ rise times* (μs) estimated for the $(Y_{0.99}Eu_{0.01})_6(BDC)_7(OH)_4(H_2O)_4$ and $(Y_{0.89}Tb_{0.10}Eu_{0.01})_6(BDC)_7(OH)_4(H_2O)_4$ MOFs under $\lambda_{exc}$ = 325 nm monitoring the $Eu^{3+}$ $^5D_0 \rightarrow {}^7F_4$ transition (698 nm, $Eu^{3+}$)

| Temperature | $(Y_{0.99}Eu_{0.01})_6(BDC)_7(OH)_4(H_2O)_4$ $^5D_0$ ($Eu^{3+}$) | $(Y_{0.89}Tb_{0.10}Eu_{0.01})_6(BDC)_7(OH)_4(H_2O)_4$ $^5D_0$ ($Eu^{3+}$) |
|---|---|---|
| 298 K | 4.6 ± 0.2 | 3.6 ± 0.9 |
| 323 K | 4.6 ± 0.2 | 2 ± 1 |
| 373 K | 4.5 ± 0.2 | 3.9 ± 0.8 |
| 398 K | 4.5 ± 0.2 | 6 ± 2 |
| 423 K | 4.5 ± 0.2 | 6 ± 3 |
| 473 K | 4.6 ± 0.2 | 2 ± 2 |
| 523 K | 4.3 ± 0.1 | 23 ± 3 |
| 573 K | 4.1 ± 0.1 | 38 ± 3 |

*(* Rise times were estimated by fitting the rise in intensity at the beginning of the intensity vs. time curves in Figure S21 by a monoexponential function, yielding a negative pre-exponential factor; the rise times correspond to the characteristic exponential constants. The large errors for $(Y_{0.89}Tb_{0.10}Eu_{0.01})_6(BDC)_7(OH)_4(H_2O)_4$ at 298-473 K stem from the low number of points available to fit the rise in intensity, as observed in Fig S21d)*

**2.8. Reversibility of decay times towards $D_2O$ exposure**

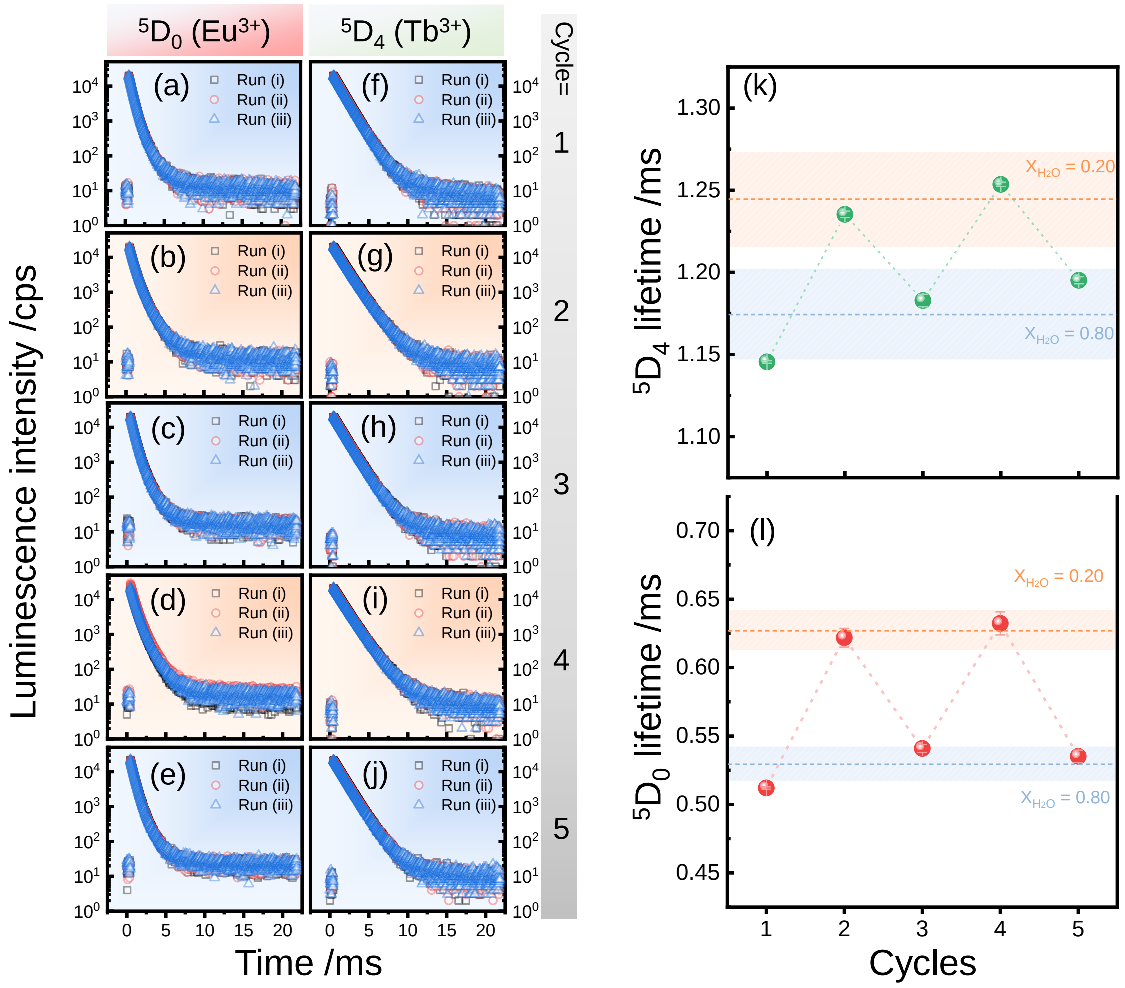


**Figure S22.** Reversibility of luminescence lifetimes of $Eu^{3+}$ ($^5D_0$) and $Tb^{3+}$ ($^5D_4$) in the $(Y_{0.99}Eu_{0.01})_6(BDC)_7(OH)_4(H_2O)_4$ MOF as 3 mg $mL^{-1}$ aqueous suspensions against variations on the $H_2O/D_2O$ molar fraction [$H_2O/D_2O$=80/20 ($X_{H2O}$=0.8) or $H_2O/D_2O$=20/80 mol/mol ($X_{H2O}$=0.2)]. (a-j) Luminescence decay profiles ($\lambda_{exc}$=325 nm) of the (a-e) $Eu^{3+}$ $^5D_0$ state ($\lambda_{em}$=698 nm) and (f-j) $Tb^{3+}$ $^5D_4$ state ($\lambda_{em}$=544 nm) for suspensions in (a/f, c/h, e/j) $X_{H2O}$=0.8 and (b/g, d/i) $X_{H2O}$=0.2 after five cycles; each cycle comprises a triplicate of runs [*i.e.*, (i), (ii) and (iii)] monitoring $Eu^{3+}$ or $Tb^{3+}$ emissions. (k,l) Reversibility of lifetimes of (k) $^5D_4$ ($Tb^{3+}$) and (l) $^5D_0$ ($Eu^{3+}$) emitting states after five cycles alternating the composition of the solvent between $X_{H2O}$=0.8 and $X_{H2O}$=0.2. The lifetimes were calculated by fitting the decay curves by mono- ($Tb^{3+}$) and bi-exponential ($Eu^{3+}$) functions. The points and error bars correspond respectively to the arithmetic means and standard deviations within each triplicate. The horizontal dashed lines and highlighted regions indicate the average lifetime values for $X_{H2O}$=0.8 (blue) and $X_{H2O}$=0.2 (orange) within an accuracy of 2.5%.

The decay profiles of the $Tb^{3+}$ ($^5D_4$) and $Eu^{3+}$ ($^5D_0$) emitting states were measured in multiple cycles where the composition of the solvent (*i.e.*, $H_2O/D_2O$ molar fraction) was varied to attest the reversibility of the response of luminescence lifetimes of the (Y,Tb,Eu)$_6$-MOF to $D_2O$. For the first cycle, 3 mg mL$^{-1}$ suspensions of the (Y,Tb,Eu)$_6$-MOF in $H_2O$ and in $D_2O$ were prepared separately and mixed to afford a $H_2O/D_2O$ molar ratio of 80/20 ($X_{H2O}$=0.8). The decay profiles the $^5D_4$ ($\lambda_{em}$=544 nm) and $^5D_0$ ($\lambda_{em}$=698 nm) emitting states were measured under $\lambda_{exc}$=325 nm excitation after ~2 min homogenisation. The decay profiles of $Tb^{3+}$ and $Eu^{3+}$ were then recorded three times. For the second cycle, the suspension was centrifuged (10000 rpm, 3 min), and the resulting solid was resuspended in a $H_2O/D_2O$=20/80 (mol/mol) mixture to afford again a ~3 mg mL$^{-1}$ concentration of the (Y,Tb,Eu)$_6$-MOF. Then, the $^5D_4$ and $^5D_0$ decay times were measured again three times using the same excitation/emission wavelengths. The centrifugation/resuspension procedure was repeated other 3 times alternating the solvent composition between $H_2O/D_2O$=80/20 ($X_{H2O}$=0.8) and $H_2O/D_2O$=20/80 ($X_{H2O}$=0.2), and measuring the $^5D_4$ and $^5D_0$ decay profiles (Figures S22a-j). In all cases, the three runs yielded nearly identical intensity vs. time curves and resulted in negligible standard deviations for characteristic lifetimes within each triplicate. Because lifetimes remain constant for three runs in each cycle after ~2 min of homogenisation, our results suggest that the OH/OD exchange in the (Y,Tb,Eu)$_6$-MOF after $H_2O$ or $D_2O$ exposure reach a quasi-equilibrium state after few minutes at 298 K. The variation of the $^5D_4$ and $^5D_0$ lifetimes upon consecutive inversions of the $H_2O/D_2O$ proportion (Figures 22k and 22l) demonstrate the reversibility of the luminescent response to $D_2O$. The lifetimes are reversible towards alterations from $X_{H2O}$=0.8 and $X_{H2O}$=0.2 within ±2.5% of the average lifetime values for each solvent composition. The minor alterations in lifetimes observed along the cycles occur because (i) the (Y,Tb,Eu)$_6$-MOF powder recovered after centrifugation was not dried before the next cycle, so a residual amount of the solvent of the previous step was always present; (ii) the centrifugation/resuspension process result in slight losses of suspended material, thus resulting in a minor decrease of the MOF concentration.

## 2. REFERENCES


(1) Larson, A. C.; Dreele, R. B. V., *Los Alamos National Laboratory Report LAUR,* **2000**, *86-748.*

(2) Cui, Y.; Xu, H.; Yue, Y.; Guo, Z.; Yu, J.; Chen, Z.; Gao, J.; Yang, Y.; Qian, G.; Chen, B. A luminescent mixed-lanthanide metal-organic framework thermometer. *J. Am. Chem. Soc.* **2012**, *134*, 3979–3982.

(3) Rao, X.; Song, T.; Gao, J.; Cui, Y.; Yang, Y.; Wu, C.; Chen, B.; Qian, G. A highly sensitive mixed lanthanide metal-organic framework self-calibrated luminescent thermometer. *J. Am. Chem. Soc.* **2013**, *135*, 15559–15564.

(4) Cadiau, A.; Brites, C. D. S.; Costa, P. M. F. J.; Ferreira, R. A. S.; Rocha, J.; Carlos, L. D. Ratiometric nanothermometer based on an emissive $Ln^{3+}$-organic framework. *ACS Nano* **2013**, *7*, 7213–7218.

(5) N'Dala-Louika, I.; Ananias, D.; Latouche, C.; Dessapt, R.; Carlos, L. D.; Sarier-Brault, H. Ratiometric mixed Eu-Tb metal-organic framework as a new cryogenic luminescent thermometer. *J. Mater. Chem. C* **2017**, *5*, 10933-10937.

(6) Cui, Y.; Zou, W.; Song, R.; Yu, J.; Zhang, W.; Yang, Y.; Qian, G. A ratiometric and colorimetric luminescent thermometer over a wide temperature range based on a lanthanide coordination polymer. *Chem. Commun.* **2014**, *50*, 719–721.

(7) Han, Y. H.; Tian, C. Bin; Li, Q. H.; Du, S. W. Highly chemical and thermally stable luminescent $Eu_xTb_{1-x}$ MOF materials for broad-range pH and temperature sensors. *J. Mater. Chem. C* **2014**, *2*, 8065–8070.

(8) Liu, X.; Akerboom, S.; de Jong, M.; Mutikainen, I.; Tanase, S.; Meijerink, A.; Bouwman, E. Mixed-lanthanoid metal-organic framework for ratiometric cryogenic temperature sensing. *Inorg. Chem.* **2015**, *54*, 11323–11329.

(9) Liu, W.; Liu, L.; Wang, Y.; Chen, L.; Mcleod, J. A.; Yang, L.; Zhao, J.; Liu, Z.; Diwu, J.; Chai, Z.; Albrecht-Schimitt, T. E.; Liu, G.; Wang, S. Tuning mixed-valent $Eu^{2+}/Eu^{3+}$ in strontium frameworks for multichannel photoluminescence formate. *Chem. - A Eur. J.* **2016**, *22*, 11170.

(10) Yang, Y.; Chen, L.; Jiang, F.; Yu, M.; Wan, X.; Zhang, B.; Hong, M. A family of doped lanthanide metal-organic frameworks for wide-range temperature sensing and tunable white light emission. *J. Mater. Chem. C* **2017**, *5*, 1981–1989.

(11) Zhao, D.; Wang, H.; Qian, G. Synthesis, structure and temperature sensing of a lanthanide-organic framework constructed from a pyridine-containing tetracarboxylic acid ligand. *CrystEngComm* **2018**, *20*, 7395–7400.

(12) Liu, J.; Pei, L.; Xia, Z.; Xu, Y. Hierarchical accordion-like lanthanide-based metal-organic frameworks: solvent-free syntheses and ratiometric luminescence temperature-sensing properties. *Cryst. Growth Des.* **2019**, *19*, 6586–6591.

(13) Pan, Y.; Su, H. Q.; Zhou, E. L.; Yin, H. Z.; Shao, K. Z.; Su, Z. M. A stable mixed lanthanide metal-organic framework for highly sensitive thermometry. *Dalton Trans.* **2019**, *48*, 3723–3729.

(14) Yang, Y.; Wang, Y.; Feng, Y.; Song, X.; Cao, C.; Zhang, G.; Liu, W. Three isostructural $Eu^{3+}$/$Tb^{3+}$ co-doped MOFs for wide-range ratiometric temperature sensing. *Talanta* **2020**, *208*, 120354.

(15) Brites, C. D. S.; Millán, A.; Carlos, L. D. Lanthanides in Luminescent Thermometry. In *Handbook on the Physics and Chemistry of Rare Earths*; Bünzli, J.-C. G., Pecharsky, V. K., Eds.; Elsevier: Amsterdam, 2016; Vol. 49, pp 339–427.
